# Replacement of Volatile Acetic Acid by Solid SiO_2_@COOH Silica (Nano)Beads for (Ep)Oxidation Using Mn and Fe Complexes Containing BPMEN Ligand

**DOI:** 10.3390/molecules26185435

**Published:** 2021-09-07

**Authors:** Yun Wang, Florence Gayet, Jean-Claude Daran, Pascal Guillo, Dominique Agustin

**Affiliations:** 1 CNRS, LCC (Laboratoire de Chimie de Coordination), Université de Toulouse, UPS, INPT, 205, Route de Narbonne, F-31077 Toulouse, France; thomaswang1990@hotmail.com (Y.W.); florence.gayet@ensiacet.fr (F.G.); jean-claude.daran@lcc-toulouse.fr (J.-C.D.); 2 Département de Chimie, Institut Universitaire de Technologie Paul Sabatier, Université de Toulouse, Av. Georges Pompidou, BP 20258, CEDEX, F-81104 Castres, France; 3 INPT, École Nationale Supérieure des Ingénieurs en Arts Chimiques et Technologiques, CS 44362, CEDEX 4, F-31030 Toulouse, France

**Keywords:** manganese complexes, iron complexes, oxidation, epoxidation, functionalized silica beads, H_2_O_2_, replacement of volatile reagent

## Abstract

Mn and Fe BPMEN complexes showed excellent reactivity in catalytic oxidation with an excess of co-reagent (CH_3_COOH). In the straight line of a cleaner catalytic system, volatile acetic acid was replaced by **SiO_2_** (nano)particles with two different sizes to which pending carboxylic functions were added (**SiO_2_@COOH**). The **SiO_2_@COOH** beads were obtained by the functionalization of SiO_2_ with pending nitrile functions (**SiO_2_@CN**) followed by CN hydrolysis. All complexes and silica beads were characterized by NMR, infrared, DLS, TEM, X-ray diffraction. The replacement of CH_3_COOH by **SiO_2_@COOH** (100 times less on molar ratio) has been evaluated for (ep)oxidation on several substrates (cyclooctene, cyclohexene, cyclohexanol) and discussed in terms of activity and green metrics.

## 1. Introduction

The synthesis of epoxides/ketones is an interesting research field from the fundamental to the applicative point of view in organic synthesis or catalysis. Indeed, those organic compounds can be obtained using very simple organic oxidants (but quite tedious in the post-treatment procedure) like meta-chloroperbenzoic acid (m-CPBA) [1,2], NaIO_4_ [3], RCO_3_H [4,5,6]. They can also be obtained using metal catalysts and the use of an organic solvent is very often required [7,8,9]. It can be the case with several Mo complexes [10,11,12,13,14]. The use of chlorinated solvents such as dichloroethane (DCE), a highly toxic solvent, has to be avoided [15]. In the research group, the processes have been found to be active without organic solvent using complexes with tridentate ligands [16,17,18,19,20] or polyoxometalates (POMs) [21,22,23], giving a first step towards a cleaner process. The oxidant used in this case is *tert*-butyl hydroperoxide (TBHP) in aqueous solution. In terms of atom economy, the epoxidation reaction could be improved using H_2_O_2_ as the oxidant. Selective epoxidation reactions were achieved using (BPMEN)Mn(OTf)_2_ [24,25,26], (BPMEN)Fe(OTf)_2_ or (Me_2_PyTACN)Fe(OTf)_2_ [27,28,29,30,31,32,33,34,35] as catalysts (BPMEN = N,N′-dimethyl-N,N′-bis(pyridin-2-ylmethyl)ethane-1,2-diamine, Me_2_PyTACN = 1,4-dimethyl-7-(2-pyridylmethyl)-1,4,7-triazacyclononane), using H_2_O_2_ as oxidant in acetonitrile as the organic solvent with high selectivity towards epoxides when acetic acid is added as co-reagent [36,37]. Indeed, by blocking one of the two labile sites on the metal center, the access to *cis*-diols is not possible [36,37]. Moreover, acting as a proton relay, the carboxylic acid protonates the distal oxygen of the metal-hydroperoxo intermediate, favoring the heterolytic O-O bond cleavage and leading to the clean formation of a metal-oxo compound, an intermediate responsible for the selective oxidation of the olefin into epoxide [37,38]. When BPMEN is used as ligand, a high quantity of acetic acid is used (14 equiv. vs. substrate), with a volume comparable to the one of the organic solvent engaged in the reaction. An elegant way to replace the organic volatile carboxylic acid by recoverable objects could be the use of a solid reagent with COOH pending functions [39,40,41,42]. For this, it was interesting to use the possibility of the functionalization of silica—using trialkoxysilane precursors—to obtain pending acidic functions on silica [43,44,45,46]. Silica was employed previously for different uses, especially to graft, in a covalent way, polydentate ligands and related complexes for catalyzed reactions, or to trap heavy metals for depollution concerns. Those strategies used mainly mesoporous compounds [47,48,49,50,51] but rarely nonporous silica beads. Few examples are related to the replacement of carboxylic function in oxidation reactions catalyzed by Fe or Mn complexes surrounded by tetradentate ligands. Notestein and coworkers reported mono- or di-nuclear Mn complexes of Me_3_tacn (1,4,7-Trimethyl-1,4,7-triazacyclononane) partially grafted on functionalized mesoporous silica with pendant carboxylic functions. The functions could recover catalyst and replace volatile reagents. Those systems showed interesting results in the oxidation reaction on several substrates [52,53].

In order to find a nonvolatile acidic agent, we used COOH functionalized silica beads instead of acetic acid. To prove the efficiency, the (ep)oxidation reactions were performed with several metal complexes based on BPMEN ligands. Although those metal complexes are not the most efficient for oxygen atom transfer (OAT) reactions, they are advantageous for a proof of concept. Well described in the literature [29,54,55] and with straightforward synthesis, [29] they have well-reported OAT reactivity [55]. The effect of the metal and/or counterion of the catalysts was studied herein. The quantity of COOH functions was evaluated according to the size of the synthesized silica beads. From the results, the green metrics have been used to compare the different methods.

## 2. Results and Discussion

### 2.1. Metal Complexes

#### 2.1.1. Synthesis

**Figure 1:**
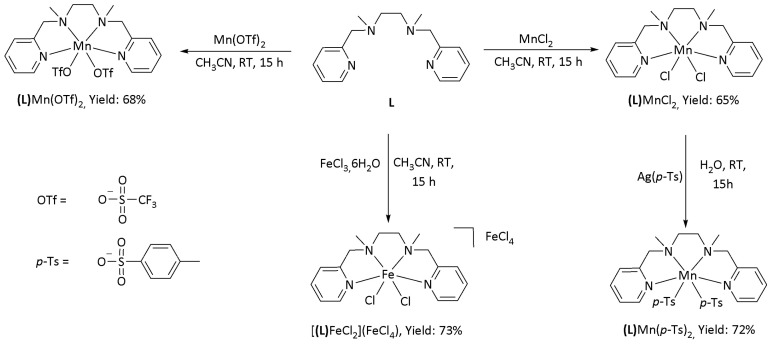
Synthesis of metal complexes of **L**.

In order to study the influence of the counter anion during the catalysis and more particularly with the use of the silica beads, three Mn^II^ metal complexes with different anions were synthetized according to Figure 1. (**L**)MnCl_2_ was obtained in 65% yield by reaction between BPMEN (**L**) and MnCl_2_·4H_2_O in acetonitrile [56]. Similarly, (**L**)Mn(OTf)_2_ was obtained in 68% yield [29]. (**L**)Mn(p-Ts)_2_ was obtained from (**L**)MnCl_2_ via anion metathesis using silver *para*-toluenesulfonate. Precipitation of AgCl during the reaction confirmed the anion exchange and (**L**)Mn(*p*-Ts)_2_ was isolated in 72% yield.

One Fe^III^ metal complex, [(**L**)FeCl_2_](FeCl_4_), determined by X-ray analysis (vide infra), was obtained in 73% yield by reaction between **L** and 2 equivalents of FeCl_3_·6H_2_O in acetonitrile. It has to be noted that the same reactivity has been observed with other ligands in the literature [57,58].

#### 2.1.2. X-ray Characterization of the Complexes

**Figure 2:**
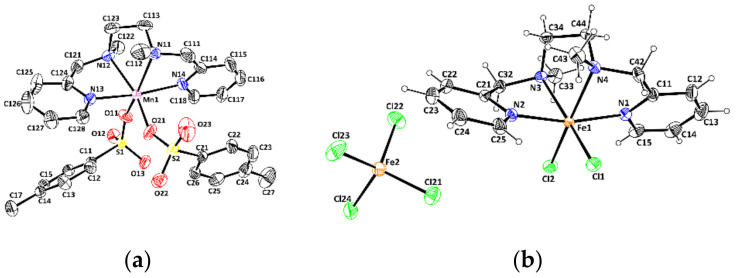
Molecular views of (**L**)Mn(*p*-Ts)_2_ (**a**) and [(**L**)FeCl_2_](FeCl_4_) (**b**) with the atom labelling scheme. Ellipsoids are drawn at the 50% probability level. H atoms have been omitted for the sake of clarity for (**L**)Mn(*p*-Ts)_2_.

Suitable crystals for X-ray analysis were obtained for all four metal complexes. The X-ray structures of (**L**)MnCl_2_ [56] and (**L**)Mn(OTf)_2_ [59] have been previously described in the literature. During the X-ray analysis, the same crystallographic parameters were obtained, confirming the nature of the metal complexes described in Figure 1. Concerning (**L**)Mn(*p*-Ts)_2_ and [(**L**)FeCl_2_](FeCl_4_), their X-ray structures are represented in Figure 2, and principal bond lengths and angles listed in Table 1. Complete data are in Supplementary Materials Tables S1–S3.

In both structures, the metal center is in a distorted octahedral environment. Several ligand-metal-ligand angle values in both metal complexes deviate significantly from the ideal values of a regular octahedron. However, all the angles measured fall in the range found for similar metal complexes in the literature, notably (**L**)MnCl_2_ [56] and (**L**)Mn(OTf)_2_ [59]. The metal centers are coordinated by the four nitrogen atoms of the **L** ligand and two anions. In both cases, the two anions are in *cis* positions and the two pyridine groups of **L** *trans* to one another. Consequently, the **L** ligand folds around the metal center using the *cis*-α conformation usually observed within this family of aminopyridine ligands.

### 2.2. Silica Beads

#### 2.2.1. Synthesis

**Figure 3:**
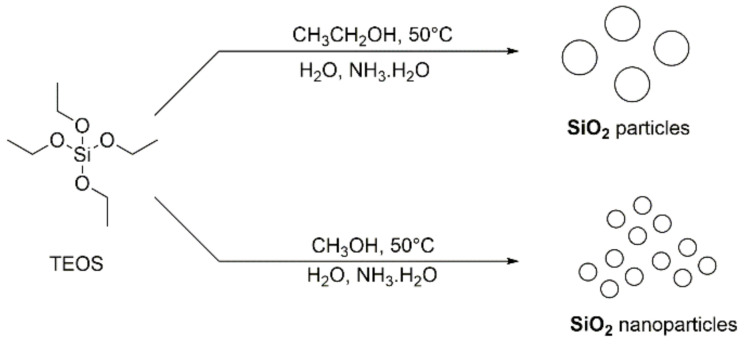
Synthesis of SiO_2_ particles.

The syntheses of **SiO_2_@COOH** (nano)particles were obtained ab initio starting from the synthesis of **SiO_2_** beads—according to a modified Stöber synthesis—using Si(OEt)_4_ (TEOS) as precursor in presence of aqueous ammonia solution and H_2_O in alcohol (ethanol or methanol) as solvent (Figure 3) [60]. The influence of solvent, [61] quantity of water, [62,63] concentration of ammonia solution [64] and temperature [65] on the size of silica nanoparticles have already been described in different articles [66]. The size of the particles decreases when solvent polarity increases [67]. Two batches of silica particles were synthesized according to the nature of solvent used during the synthesis. Their reactivity will be compared in several catalyzed oxidation reactions.

**Figure 4:**
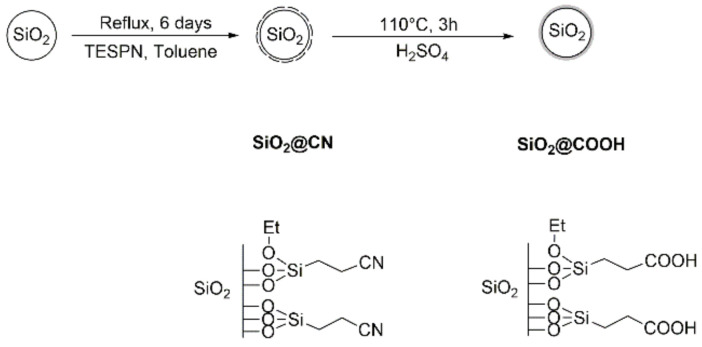
Synthetic pathway of the functionalized SiO_2_ nanoparticles.

The syntheses of **SiO_2_@COOH** were performed in two steps (Figure 4). The first step is the functionalization of the surface of the **SiO_2_** nanoparticles by 3-(triethoxysilyl)propionitrile (TESPN) in order to obtain the available nitrile functions **SiO_2_@CN**. The terminal nitrile functions were hydrolyzed in a second step into carboxylic ones using H_2_SO_4_ (65 wt.%) to obtain the **SiO_2_@COOH** beads. All (nano)particles (**SiO_2_**, **SiO_2_@CN**, **SiO_2_@COOH**) were characterized by TEM, DLS, solid NMR and the number of functions grafted quantified by solution ^1^H NMR.

#### 2.2.2. Characterization

The purpose of two different solvents for the synthesis of the starting **SiO_2_** was to access different beads sizes. Indeed, different sized nonporous silica beads might lead to different specific surfaces (linked to the average diameter of the beads) and might influence the number of grafted functions per gram of silica beads. Thus, objects of different sizes can be added into the reaction media and might change the reactivity and/or the reaction mass efficiency (RME) in the catalyzed oxidation reactions studied herein.

The morphological study of the (nano)particles was done by TEM and DLS to determine their sizes and behaviors in suspension. The proof of the grafting was done using different spectroscopic methods (IR, solid NMR) and the quantification of the grafting through ^1^H liquid NMR.

##### Morphological Study

- Transmission electron microscope (TEM) analysis

**Figure 5:**
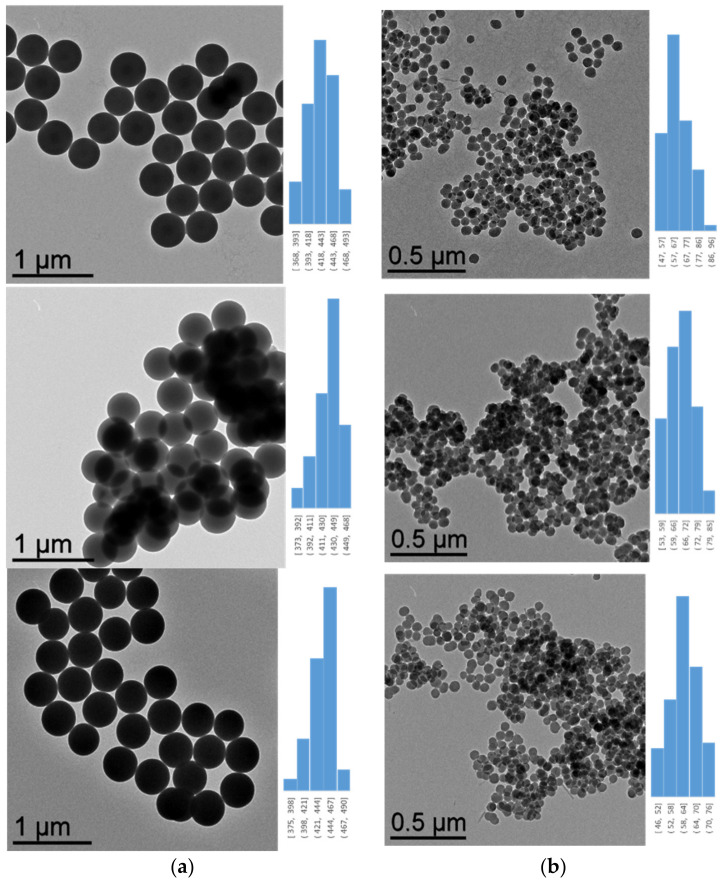
From top to bottom: TEM images and diameter distribution of **SiO_2_**, **SiO_2_@CN**, **SiO_2_@COOH** beads from SiO_2_ beads produced in EtOH (**a**) and MeOH (**b**).

From the TEM pictures in the case of the **SiO_2_**, **SiO_2_@CN** and **SiO_2_@COOH** beads (Figure 5), it has been possible to prove the size of the silica beads according to the solvent used. For each step, monodisperse spherical beads have been obtained of around 430−440 nm when produced in ethanol and 62−66 nm when produced in methanol.

- Dynamic light scattering (DLS) measurements

Monodispersity is an important parameter for **SiO_2_@CN** and **SiO_2_@COOH** beads, ensuring reproducible catalytic reactions. DLS is another practical and simple method which could determinate the hydrodynamic radius distribution of silica particles.

**Figure 6:**
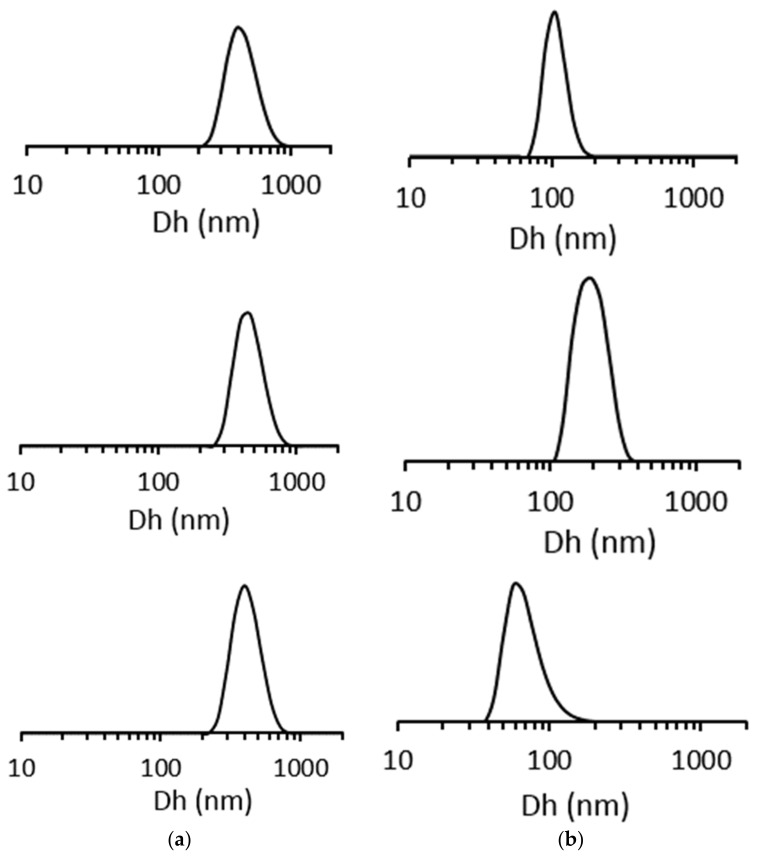
From top to bottom: size (hydrodynamic radius) distribution (in number) obtained by DLS for **SiO_2_**, **SiO_2_@CN**, **SiO_2_@COOH** beads from **SiO_2_** beads produced in EtOH (**a**) and MeOH (**b**).

DLS measurements for **SiO_2_**(E), **SiO_2_@CN**(E) and **SiO_2_@COOH**(E) (E: ethanol) show regular hydrodynamic radii of the particles around 400–450 nm, close to the ones found by TEM, especially because the grafted function thickness is small compared to the bead sizes (Figure 6). The narrow distribution confirmed the relatively monodisperse beads.

In the case of **SiO_2_**(M) (M: methanol) beads, for which the size was smaller, the DLS measurements (100 nm for **SiO_2_**, 190 nm for **SiO_2_@CN** and 68 nm for **SiO_2_@COOH**) did not give data in accordance with the observations from TEM. This could be due to some aggregation phenomena or, in the case of **SiO_2_@CN**, multilayers of silanes.

##### Spectroscopic Characterization of the Grafting

- Infrared spectroscopy

**Figure 7:**
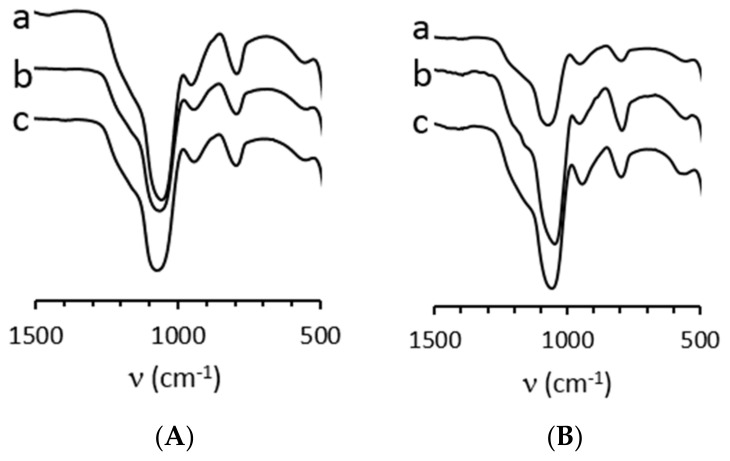
Relevant IR vibration zones for **SiO_2_** (a), **SiO_2_@CN** (b), **SiO_2_@COOH** (c) beads from **SiO_2_** beads produced in EtOH (**A**) and MeOH (**B**).

The IR spectra of all silica beads (Figure 7) showed typical vibration bands in accordance with the SiO_2_ core at 793 cm^−1^ for Si-O-Si symmetrical vibration, 945 cm^−1^ for Si-OH, 1060 cm^−1^ for Si-O-Si asymmetrical ones, 3700 cm^−1^-2930 cm^−1^ for -OH in stretching mode. In the case of **SiO_2_@CN** vibrations at 2250 cm^−1^ for CN [68] and 2832 cm^−1^ for CH stretching mode [69]. The presence of carboxylic functions could be detected, i.e., C=O for **SiO_2_@COOH** at 1712 cm^−1^ [70,71].

The size of the starting **SiO_2_** does give different intensities for the grafted fragments. Indeed, while it is very easy to observe the vibrations assigned to grafted organic part with the SiO_2_@f(M) beads, it is less obvious in the case of SiO_2_@f(E). This has to be linked to the grafted functions per size of beads ratio. The smaller the bead is, the “more intense” will be the vibrational pattern of the organic part.

**Figure 8:**
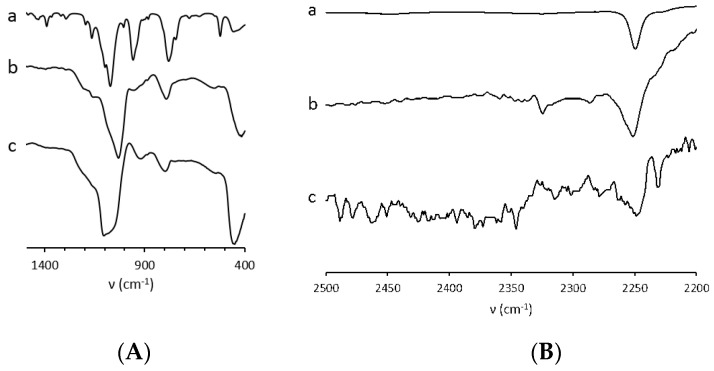
Difference spectra (**SiO_2_@CN**-**SiO_2_**) on two specific ranges, i.e 400-1500 cm^−1^ (**A**) and 2200-2500 cm^−1^ (**B**). The spectrum of TESPN is indicated in (a), (b) **SiO_2_** produced in MeOH, (c) with **SiO_2_** produced in EtOH.

**Figure 9:**
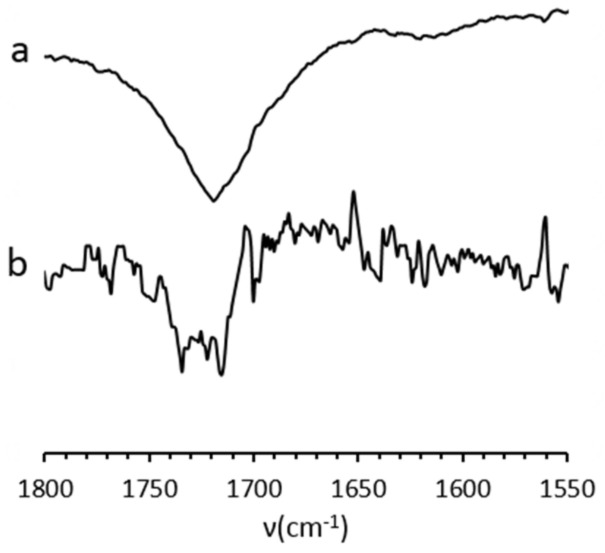
Difference spectra (**SiO_2_@COOH**-**SiO_2_**) on specific range. (a) with **SiO_2_** produced in MeOH, (b) with **SiO_2_** produced in EtOH.

Due to low loading of the grafted functions in the case of **SiO_2_@CN**(E) and even lower in **SiO_2_@COOH**(E) because of the acid hydrolysis, the vibrations corresponding to functional groups were observed with difficulty from the raw spectra. Those vibrations that could be seen were giving difference spectra between **SiO_2_@CN** and **SiO_2_** OR between **SiO_2_@COOH** and **SiO_2_**, proving the existence of the -CN (Figure 8) and -COOH (Figure 9) functional groups.

- Solid state NMR

To increase the knowledge about grafting, the multinuclear solid state (CP)MAS NMR (^1^H, ^13^C and ^29^Si) can be investigated. All data have been summarized in Supplementary Materials Table S4. All relevant information will be discussed through nuclei.

The ^1^H MAS NMR’s very large (and sometimes overlapped) signals are indicative and correspond to different groups on the silica beads, i.e., silanols and physiosorbed water molecules (3.5–5 ppm), EtO (3.3–3.6 ppm), MeO (1.1–1.3 ppm) groups as well as CH_2_ from the grafted units (0.7–0.9 (Si-CH_2_), 6.5–6.8 (CH_2_-N) 4.0–4.1 (CH_2_)) [72].

The ^13^C CP-MAS NMR spectra show signals corresponding to the organic functions grafted on **SiO_2_**. EtO functions are present in both **SiO_2_** starting beads and after grafting. The signals corresponding to the silane with CN are visible with **SiO_2_@CN**, as well as with COOH after the hydrolysis for **SiO_2_@COOH** (see Supplementary Materials Table S4 and Figure S1) [72], confirming the grafting and the transformation of the pending function.

**Figure 10:**
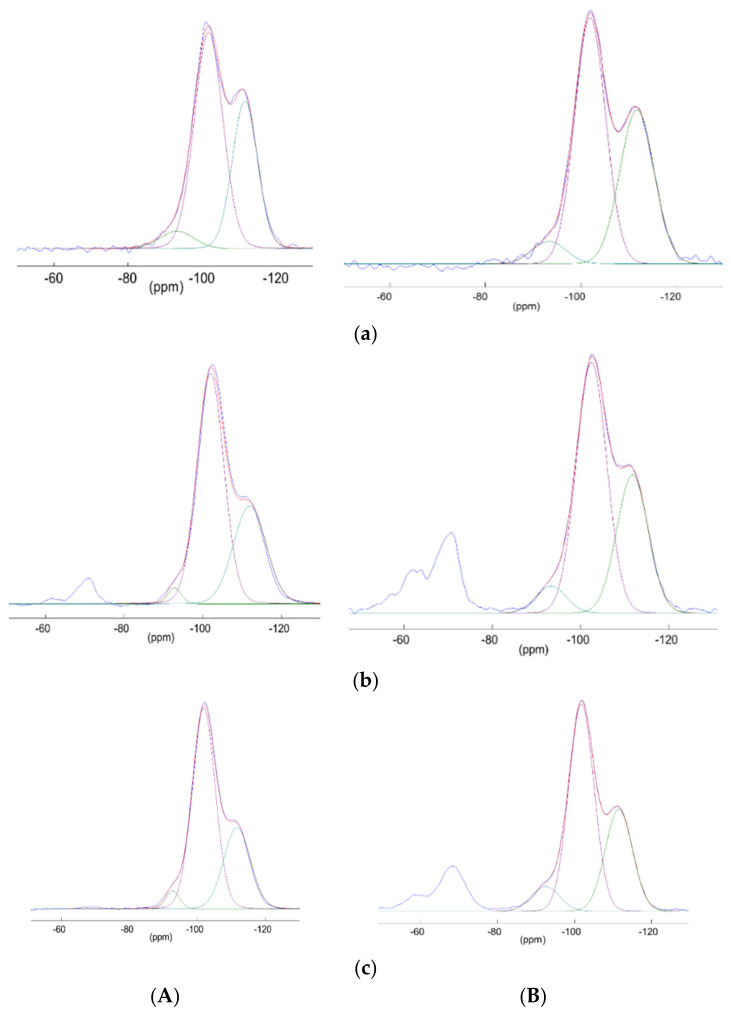
^29^Si CPMAS NMR spectra of **SiO_2_** (**a**) **SiO_2_@CN** (**b**), **SiO_2_@COOH** (**c**) from **SiO_2_** produced in EtOH (**A**) and MeOH (**B**).

The ^29^Si CP-MAS NMR spectra gave other information (Table S4 and Figure 10). In all spectra, the signals at −93, −101 and −111 ppm corresponding to Q_2_, Q_3_ and Q_4_ respectively (Q_n_= Si(OSi)_n_(OH)_4−n_) are in accordance with SiO_2_ core [73,74]. The grafting was proved by two signals at around −60 and −70 ppm (T_2_ and T_3_) [75]. A change in the proportion of the signals was observed from **SiO_2_** to **SiO_2_@CN** and from **SiO_2_@CN** to **SiO_2_@COOH**, the trend being identical with the starting **SiO_2_**(M) and **SiO_2_**(E) beads. Since CP MAS could not be used to quantify the Q_n_, the deconvolutions were performed on MAS spectra (Figure S2). The intensity distribution is summarized in Table S4. 

The solid-state NMR showed that the **SiO_2_** beads contain some ethoxy functions (although dried under vacuum) and those functions remain even when the grafting occurs. ^29^Si NMR spectra exhibit a qualitative change of the silicon core with the grafted functions. In order to use those beads in a precise and quantitative manner, it was important to quantify the grafted functions at the surface through different parameters.

- Quantification by ^1^H NMR in solution

When an analyzed sample is simple or pure, elemental analysis (EA) can give accurate information. In the case of the presented silica beads, the system—as shown by multinuclear MAS NMR—is more complex and EA would not give reliable results. One elegant method has been developed [40], considering that, in a very alkaline medium, silica can be transformed into silicates maintaining the integrity of the organic fragments that can be easily quantified by ^1^H solution NMR, using an internal standard (benzoic acid herein, stable and soluble in very basic solution as benzoate).

Thus, a mass of sample silica beads was dissolved in strong alkaline deuterated aqueous solution (pH ≈ 13) and analyzed by ^1^H NMR using a mass of internal standard, giving a number of moles of functions per gram of silica beads (all beads, i.e., **SiO_2_**, **SiO_2_@CN** and **SiO_2_@COOH**).

**Figure 11:**
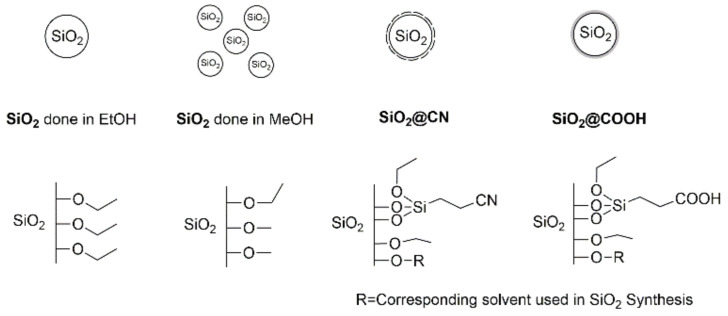
Schematic functions on the silica beads.

The signals corresponding to ethanol and methanol are related to the alkoxy functions present on beads, from TEOS to TESPN (Figure 11). All the other CH_2_ signals are related to the non-alkoxy part of TESPN and the corresponding oxidized one. The ^1^H NMR shifts have been presented in Table S5.

The number of functions n(f) has been calculated based on ^1^H NMR integrations I(f) relatively to I(ref) from a known mass of internal standard, m(ref) (Table 2). With n(f), the density of f functions per mass of sample ρ(f) was defined according to the mass of SiO_2_ sample (m_S_) using Equation (1).

(1) $$\rho \left(f\right)=\frac{n\left(f\right)}{m_{S}}=\frac{I\left(f\right)}{m_{S}}\cdot \frac{m\left(\mathrm{ref}\right)}{M\left(\mathrm{ref}\right)}\cdot \frac{1}{I\left(\mathrm{ref}\right)}$$

The results showed that -OEt fragments were present on starting **SiO_2_**, with a higher content per gram of sample with **SiO_2_**(M) beads (smaller size) [76,77]. The functionalization was, for the same reason, higher per gram of sample in the case of **SiO_2_@CN**(M). From **SiO_2_@CN** to **SiO_2_@COOH**, the hydrolysis removed a substantial part of the “grafted” functions, certainly destroyed/removed by concentrated sulfuric acid.

- Determination of function coverage of functionalized silica beads

**Figure 12:**
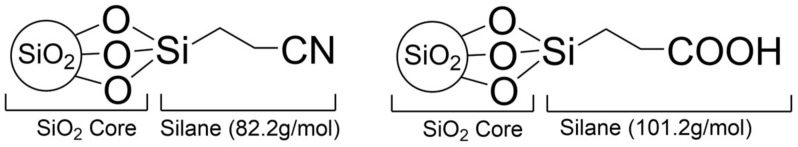
Schematic representation of the silica beads

Using several techniques, it is possible to calculate the function coverage on silica cores, an important parameter within the catalytic part. The parameter μ(f), defined in the number of groups per nm^2^, could be determined by Equation (3) [23,40]. The ρ’(f) parameter does correspond to the functions grafted on a silica core (Figure 12 and Equation (2)) and is calculated from ρ(f). The average radius of the **SiO_2_** beads (r_core_) is deduced from the TEM measurements. μ(f) was calculated with a core mass (m_core_) of 1 g.

(2) $$\rho \text{’}\left(f\right)=\frac{n\left(f\right)}{m_{\mathrm{core}}}=\frac{\rho \left(f\right)}{1-\rho \left(f\right).M_{\begin{array}{c} Silane \end{array}}}.$$

The parameter μ(f) is the number of molecules n(f) grafted on 1 g of the sample surface ΣScore (in nm^2^). From the **SiO_2_** radii found in TEM measurements, Equation (3) can be written as follows:

(3) $$\mu \left(f\right)=\frac{\rho \text{’}\left(f\right).r_{\mathrm{core}}.\rho_{{\mathrm{SiO}}_{2}}}{3.10^{+21}}\times N_{A}\;$$

Using Equation (3), coverage by CN and COOH fragments have been calculated (Table 3). Concerning the **SiO_2_@CN**, the μ(CN) value is very high (>17) and seems to confirm a multilayer deposition. The μ(COOH) values around 3 for **SiO_2_@COOH** are in agreement with what is expected with monolayers.

### 2.3. Catalysis

The BPMEN-related complexes were tested on three different substrates and two different co-reagents, CH_3_COOH (in order to use the results as reference) or **SiO_2_@COOH**. The catalytic study presented herein will be divided according to the substrates. 

The complexes were tested as homogenous catalysts under the classical conditions (using acetic acid as co-reagent) and the influence of the metal and anion was studied. The reactivity was compared with the processes using **SiO_2_@COOH** beads or acetic acid. These complexes were tested in olefin epoxidation and alcohol oxidation. For this reason, cyclooctene (CO) was chosen as model substrate for epoxidation, while the (ep)oxidation of cyclohexene (CH) and oxidation of cyclohexanol (CYol) were studied for their potential applied interest towards the synthesis of adipic acid, both being starting reagents in different processes [31,32,33,34,35,78,79].

Reaction under homogeneous conditions was previously described [31,80]. To prevent H_2_O_2_ disproportionation [81] and Fenton reaction [82], H_2_O_2_ was slowly added at 0°C for two hours [83] (especially in the case of Fe complex) [84] using CH_3_CN as solvent. The cat/substrate/H_2_O_2_/CH_3_COOH ratio of 1/100/150/1400 was followed. The reactions were stopped after 3 h and analysed by GC-FID using acetophenone as an internal standard.

#### 2.3.1. Oxidation of Cyclooctene

Cyclooctene (CO) was used as the model since the substrate is known to give the corresponding cyclooctene oxide (COE) with high selectivity. To prove the need of carboxylic function as co-reagent in this catalysis, some tests with complexes were done in the absence and presence of co-reagent (Table 4). While no CO conversion was observed with [(**L**)FeCl_2_](FeCl_4_), all (**L**)MnX_2_ complexes (X = Cl, OTf, *p*-Ts) were poorly active, showing the necessity of a carboxylic co-reagent. All complexes were tested in the presence of a co-reagent, acetic acid or **SiO_2_@COOH** (taking into account the bead sizes) under identical experimental conditions.

**Figure 13:**
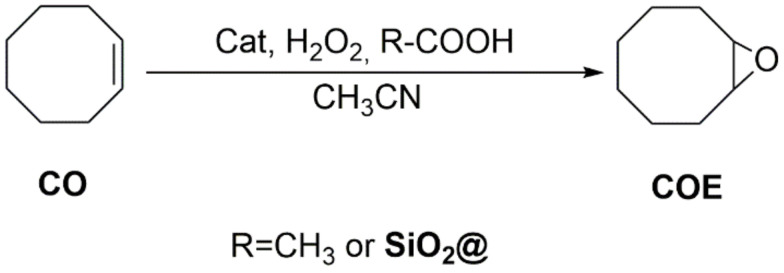
Catalytic oxidation of cyclooctene.

**Figure 14:**
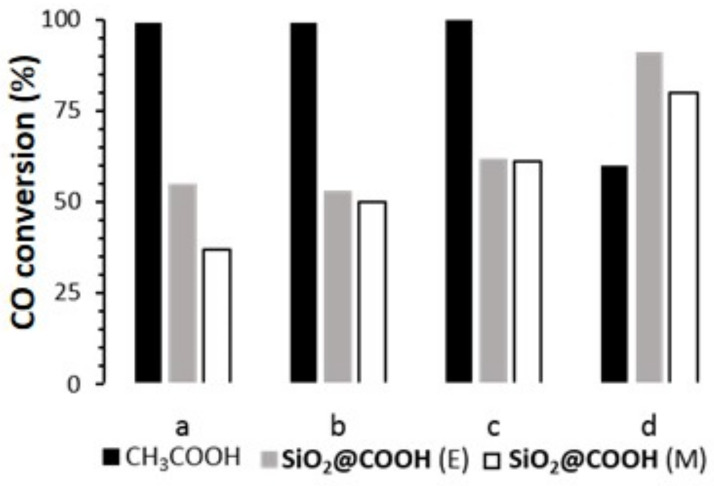
Comparison of CO conversion between different conditions for (**L**)MnCl_2_ (a), (**L**)Mn(OTf)_2_ (b), (**L**)Mn(*p*-Ts)_2_ (c), (**L**)FeCl_2_(FeCl_4_) (d).

In the presence of a co-reagent (Figure 13), all catalysts could achieve CO conversion, the best conditions being in the presence of acetic acid for manganese complexes, while the conversion was better in the presence of **SiO_2_@COOH** with the iron complex (Table 4 and Figure 14). The lower conversion in the presence of **SiO_2_@COOH** beads for manganese complexes seems to be due to the heterogeneous character of the reaction. COE was the only product observed by GC-FID. The low selectivity towards COE in the presence of (**L**)MnX_2_ (X = OTf, *p*-Ts) and [(**L**)FeCl_2_](FeCl_4_) might be due to the formation of cyclooctanediol and the subsequent opening ring reaction conducting to suberic acid [85,86]. Those two products could not be observed by GC-FID using the method developed herein.

Using CH_3_COOH as the co-reagent with a cat/CH_3_COOH ratio of 1:1400 (Table 4 and Figure 14), the results for the complexes (**L**)MnX_2_ (X = Cl, OTf) were similar to those described [29]. The manganese complexes (**L**)MnX_2_ (X = Cl, OTf, *p*-Ts) gave almost complete CO conversion. However, the selectivity towards COE with X = OTf and *p*-Ts around 60% was lower than X = Cl (81%). It can be concluded that the anion has an influence on the selectivity towards COE. It might be due to the basicity of the anion, the chloride being the more inert. As pointed out previously, the ring opening might occur in presence of acid/base, and it was certainly what happened here. However, diminishing the cat/CH_3_COOH ratio to 1:14 for (**L**)MnCl_2_ gave similar results to the ones observed in the absence of acetic acid, underlying the necessity of a huge excess of co-reagent to achieve high conversion and selectivity with complexes based on BPMEN ligand.

Very interestingly, using **SiO_2_@COOH** beads as co reagents with a cat/COOH ratio of 1:14, the conversion of CO was observed, proving the positive effect of the silica beads functionalized with COOH even with a relatively low amount of COOH functions in the reactional mixture In addition, the use of **SiO_2_@COOH** beads as co-reagents gave in the case of the manganese complexes a reverse effect (Table 4 and Figure 13) than the one observed with acetic acid. Indeed, the conversion follows the X order *p*-Ts > OTf > Cl, with a selectivity towards COE in favor of the triflate, followed by the *p*-Ts and finally the chloride salt. The effect of the bead size is negligible in the case of the two more active complexes ((L)MnX_2_ (X = OTf, *p*-Ts)) while a stronger difference is observed with the chloride salt, giving lower selectivity towards COE. 

Concerning the iron complex, a moderate conversion and a low selectivity were observed in the presence of CH_3_COOH. With silica beads, higher conversions were obtained and the selectivities were similar to the ones with CH_3_COOH.

#### 2.3.2. Oxidation of Cyclohexene

The cyclohexene (CH) is a very interesting substrate as a starting material for the synthesis of adipic acid [22,79]. In comparison to CO, the (ep)oxidation of CH is more complex. Indeed, according to the nature of the metal used within the reaction, two oxidations are possible: allylic oxidation on sp^3^ C-H bonds and epoxidation on C=C double bond [87]. Other possible water additions and/or subsequent oxidation give a complex mixture.

**Figure 15:**
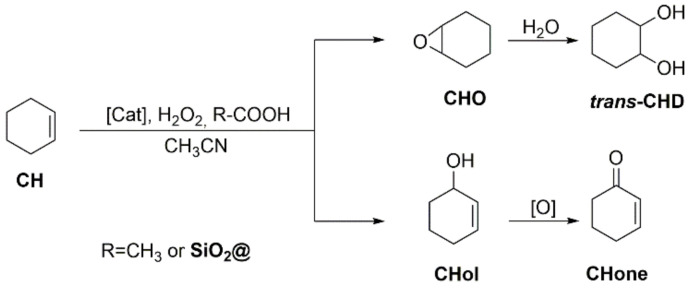
Catalytic oxidation of cyclohexene.

**Figure 16:**
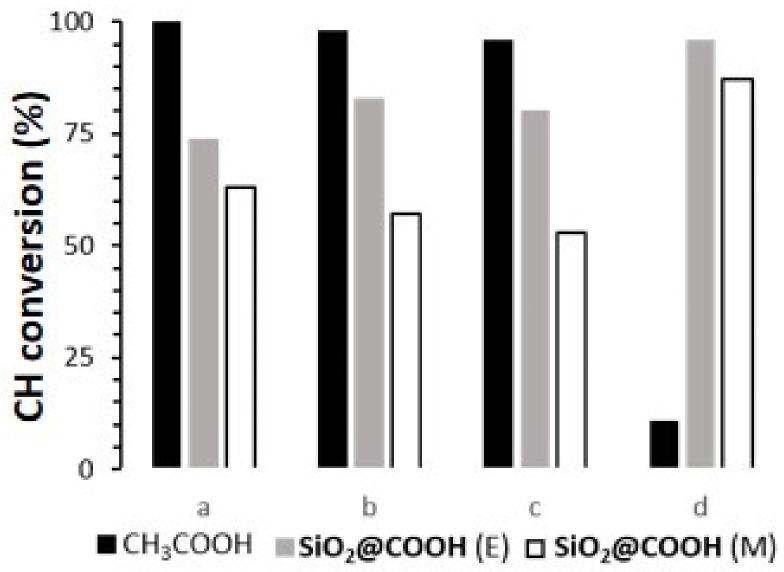
Comparison of conversion (%) of CH between different catalysts (**L**)MnCl_2_ (**a**), (**L**)Mn(OTf)_2_ (**b**), (**L**)Mn(*p*-Ts)_2_ (**c**), (**L**)FeCl_2_(FeCl_4_) (**d**) and different co-reagents. Reaction time: 3 h with CH_3_COOH, 5 h with **SiO_2_@COOH**.

Cyclooctene oxide (CHO), cyclohexanediol (CHD), cyclohexene-1-ol (CHol) and cyclohexen-1-one (CHone) are the most common observed products (see Figure 15). The conversion of CH, the selectivity towards the products and TON have been compiled (Table 5, and Figure 16).

All the manganese complexes (**L**)MnCl_2_ (X = Cl, OTf, *p*-Ts) exhibited high CH conversion in the presence of CH_3_COOH and the analysed products are anion-dependent. While X = Cl gave exclusively CHO with a relatively good selectivity (89%), the complexes with X = OTf and *p*-Ts gave a small quantity of CHD and CHone. When **SiO_2_@COOH** beads were used instead of acetic acid, the CH conversions were lower, CHO being the only product detected with X = OTf and *p*-Ts. (**L**)MnCl_2_ showed a part of ring opening (presence of CHD) with **SiO_2_@COOH**(E) beads and allylic oxidation (presence of CHol and CHone) with the **SiO_2_@COOH**(M). From those observations, it seems that the presence of CH_3_COOH or **SiO_2_@COOH** have reverse effects in terms of selectivity according to the nature of the anion of the Mn complex. This has certainly to be linked to the mechanism occurring between the manganese complex and the co-reagent linked to the nature of the interaction between the anion and the “MnL” part.

With the [(**L**)FeCl_2_]FeCl_4_ complex, the mechanism seems to be radically different since the reaction with CH_3_COOH as co-reagent gave hardly any product (although a slight conversion was observed). Surprisingly, the use of **SiO_2_@COOH** did improve the CH conversion but not in a selective way since the products originating from epoxidation and allylic oxidation were observed in almost equal quantities.

#### 2.3.3. Oxidation of Cyclohexanol

**Figure 17:**
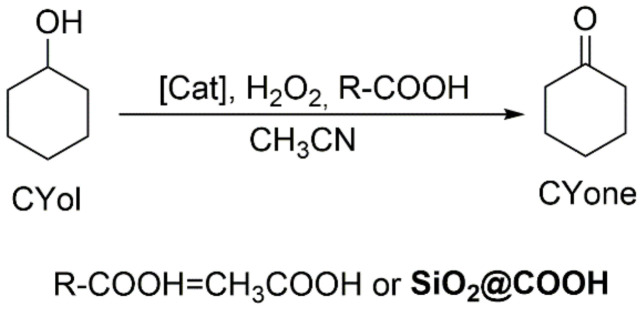
Catalytic oxidation of cyclohexanol.

**Figure 18:**
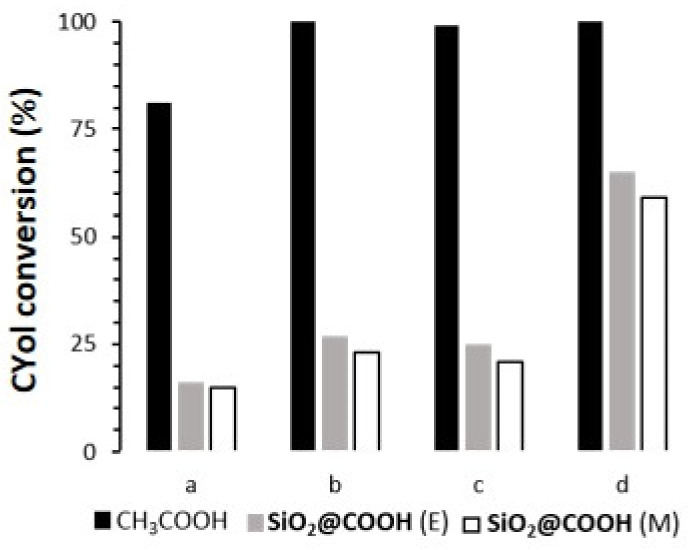
Comparison of CYol conversion (%) between different catalysts (**L**)MnCl_2_ (**a**), (**L**)Mn(OTf)_2_ (**b**), (**L**)Mn(*p*-Ts)_2_ (**c**), (**L**)FeCl_2_(FeCl_4_) (**d**) and different co-reagents. Reaction time: 3 h with CH_3_COOH, 5 h with **SiO_2_@COOH**.

The cyclohexanol (CYol) is also a very interesting substrate as a starting material of the KA oil (KA oil = ketone-alcohol oil) used for the synthesis of adipic acid [88,89]. In addition, compared to the oxidation of CH, oxidation of CYol gives only one product, i.e., cyclohexanone (CYone) (see Figure 17). Catalyzed cyclohexanol oxidation followed the same procedure as CO and CH and results have been compiled in Figure 18 and Table 6. 

With all complexes, in the presence of CH_3_COOH, the conversion of CYol was high and selective towards CYone [90,91]. (**L**)Mn(OTf)_2_ and (**L**)Mn(*p*-Ts)_2_ complexes were more active than (**L**)MnCl_2_. Due to the lability of OTf and *p*-Ts anions, the coordination site in (**L**)Mn(OTf)_2_ and (**L**)Mn(*p*-Ts)_2_ was more accessible than for (**L**)MnCl_2_. As a consequence, the access to the metal center for peroxide and carboxylic function might be favored. Due to the heterogeneous nature of the **SiO_2_@COOH** reagent, the conversion was lower in all cases. Some differences appeared in terms of selectivity, due to the nature of the anion within the complexes (in the case of the manganese complexes) and/or to the nature of the metal in the case of the iron complex. Notably, selectivity was drastically diminished for the iron complex in the presence of **SiO_2_@COOH**.

### 2.4. Green Metrics

The use of **SiO_2_@COOH** is interesting in terms of the material recovery parameter. Indeed, the studied parameter between all tests has been the replacement of acetic acid by the silica beads, and it has to be pointed out that the number of carboxylic functions is lower with the beads (from a factor 100). Some green metrics could be considered within this process [92]. The recovery of by-products (water, acetic acid and excess H_2_O_2_) would require more energy than the distillation of acetonitrile and the filtration/centrifugation of the silica beads. The difference lies, thus, in the non recovered waste.

**Figure 19:**
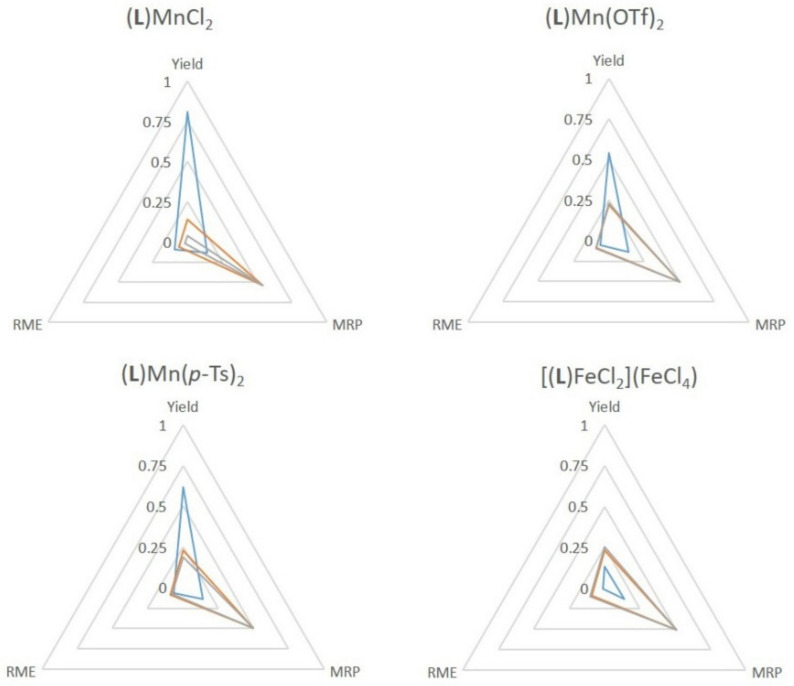
Comparison of green metrics for the epoxidation of cyclooctene (yield, RME and MRP) with the different catalysts and the different co-reagents acetic acid (blue), **SiO_2_@COOH**(E) (orange) and **SiO_2_@COOH**(M) (grey).

**Figure 20:**
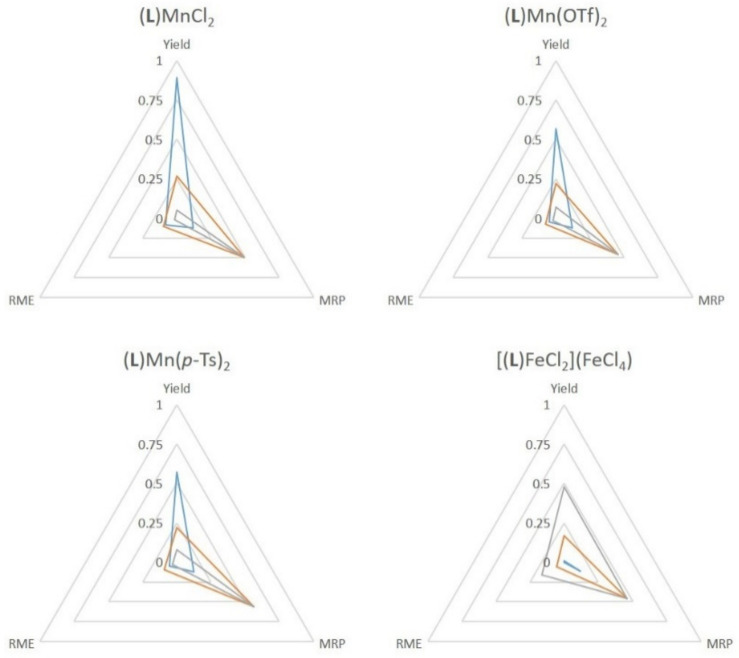
Comparison of green metrics for the epoxidation of cyclohexene (the yield considered the cyclohexene oxide only) (yield, RME and MRP) with the different catalysts and the different co-reagents acetic acid (blue), **SiO_2_@COOH**(E) (orange) and **SiO_2_@COOH**(M) (grey).

**Figure 21:**
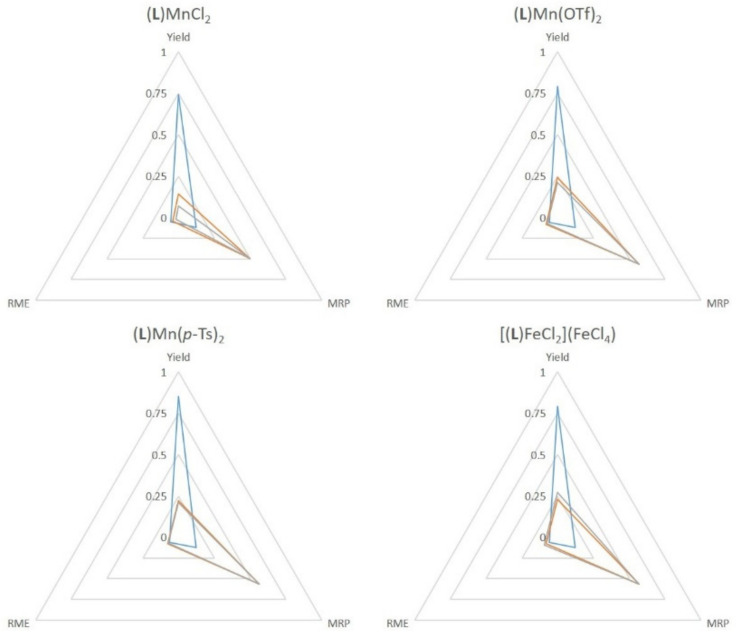
Comparison of green metrics for the oxidation of cyclohexanol (yield, RME and MRP) with the different catalysts and the different co-reagents acetic acid (blue), **SiO_2_@COOH**(E) (orange) and **SiO_2_@COOH**(M) (grey).

Considering the several green metrics, the atom efficiency (AE) and stoichiometric factors (SF)—being identical for all the studied reaction—were not added in the comparisons. The yield, the MRP and RME have been graphically presented. It can be seen that most reactions have lower yields when **SiO_2_@COOH** is used but with a slightly better RME (the mass of beads is lower than the mass of acetonitrile, even the bigger ones). The MRP is in each case in favor to **SiO_2_@COOH**. (Figure 19, Figure 20 and Figure 21). Those results represent a proof of a concept of a cleaner process.

## 3. Materials and Methods

### 3.1. Materials

All manipulations were carried out under air. Distilled water was used directly from a Milli-Q purification system (Millipore, Burlington, MA, USA). Acetonitrile, ethanol, methanol (synthesis grade, Aldrich) were used as solvents as received. Tetraethyl orthosilicate (TEOS, 98% Aldrich, St. Louis, MI, USA), ammonium hydroxide solution (25%, Aldrich), 3-(Triethoxysilyl)propionitrile (97%, Aldrich), cis-cyclooctene (95%, Alfa Aesar, Karlsruhe, Germany), cyclooctene oxide (99%, Aldrich), cyclohexene (99%, Acros), cyclohexene oxide (98%, Aldrich), 2-cyclohexen-1-ol (95%, TCI, Tokyo, Japan)), 2-cyclohexen-1-one (96%,TCI), cis-1,2-cyclohexanediol (99%, Acros, Geel, Belgium), cyclohexanol (99%, Alfa Aesar), cyclohexanone (99.8%, Acros) and TBHP (70% in water, Aldrich) were used as received.

### 3.2. Methods

#### 3.2.1. X-ray Structural Analyses

A single crystal of each compound ((**L**)Mn(*p*-Ts)_2_ and [(**L**)FeCl_2_](FeCl_4_)) was mounted under inert perfluoropolyether on the tip of a glass fiber and cooled in the cryostream of a Bruker Nonius CAD4 APEXII diffractometer. The structures were solved by using the integrate space-group and crystal structure determination SHELXT software [93] and refined by least squares procedures on F^2^ using SHELXL-2014 [94]. The crystal and refinement parameters of all compounds are collected in Table S1 and the full list of bond distances and angles provided in Supplementary Materials Tables S2 and S3. All H atoms attached to carbon were introduced in calculation in idealized positions and treated as riding models. The drawing of the molecules was realized with the help of ORTEP32 [95,96]. CCDC 1959449 (for (**L**)Mn(*p*-Ts)_2_) and 1959450 (for [(**L**)FeCl_2_](FeCl_4_)) contain the supplementary crystallographic data for this paper. These data can be obtained free of charge from The Cambridge Crystallographic Data Centre.

#### 3.2.2. Dynamic Light Scattering

Preparation sample: in order to be able to obtain repetitive and correct data analysis, particle samples were prepared at 0.1 wt.% in water. A sonication of the particles suspension was made before DLS analysis for 5 min at 350 W (FB705 Fisherbrand Ultrasonic Processor), facilitating the dispersion of silica particles. Hydrodynamic diameters of the particles in suspension were obtained with a ZetaSizer Nano-ZS (Malvern Instruments Ltd.). This equipment uses a laser (He-Ne at λ = 633 nm, under voltage of 3 mV) and the detector is located at 173° to analyse the scattered intensity fluctuations. A portion of 10 mg of particles was dispersed in 20 mL of water with the ultrasonic processor 40 (5 min, 350 W) prior to the measurement performed at a temperature of 25 °C.

#### 3.2.3. TEM

Particle morphology was performed with a JEOL JEM1011 transmission electron microscope equipped with 100 kV voltage acceleration and tungsten filament (Service Commun de Microscopie Electronique TEMSCAN, Centre de Microcaractérisation Raimond Castaing, Toulouse, France). A drop of sonicated particle solution (0.1 wt.% in ethanol) was disposed on a formvar/carbon-coated copper grid (400 mesh) and dried in air for 48 h.

#### 3.2.4. Infrared Spectroscopy

Fourier Transform infrared (FTIR) spectra were recorded by Spectrum two—PerkinElmer.

#### 3.2.5. Solid State NMR

NMR experiments were recorded on Bruker Avance 400 III HD spectrometers operating at magnetic fields of 9.4 T. Samples were packed into 4 mm zirconia rotors. The rotors were spun at 8 kHz at 293 K. ^1^H MAS was performed with DEPTH pulse sequence and a relaxation delay of 3 s. For ^29^Si MAS single pulse experiments, small flip angle of 30° was used with recycle delays of 60 s. ^13^C CP and ^29^Si CP MAS spectra were recorded with a recycle delay of 2 s and contact times of 3 ms and 4 ms, respectively. Chemical shifts were referenced to TMS. All spectra were fitted using the DMfit software.

#### 3.2.6. Solution NMR

^1^H-NMR and ^13^C-NMR spectra were recorded on Bruker NMR III HD 400 MHz spectrometers, 400 MHz for ^1^H-NMR, and 101 MHz for ^13^C-NMR. 

#### 3.2.7. Elemental Analysis

Elemental analyses were performed by the microanalysis service of the LCC.

#### 3.2.8. Centrifugation

The silica beads were collected by centrifugation on a Fisher 2-16P with 11192 rotor (Max. rpm 4500, Sigma).

#### 3.2.9. Gas Chromatography

The catalytic reactions were followed by gas chromatography on an Agilent 7820A chromatograph equipped with an FID detector, a DB-WAX capillary column (30 m × 0.32 mm × 0.5 μm) and autosampler. Authentic samples of reactants (cyclooctene, cyclohexene, cyclohexanol) and some potential products (cyclooctene oxide, cyclohexene oxide, 2-cyclohexen-1-ol, cis-1.2-cyclohexanediol, 2-cyclohexen-1-ol, and cyclohexanone) were used for calibration. The conversion and the formation were calculated from the calibration curves (r^2^ = 0.999) and an internal standard.

#### 3.2.10. Quantification of the Number of Functions per Gram of Grafted Silica through ^1^H NMR in Solution

A sample of 7 mg of SiO_2_@R (R= CN, COOH) was added to 4 mL of D_2_O/NaOH solution (pH ≈ 13) in an NMR tube. The mixture was heated until the powder completely dissolved. A known amount of benzoic acid (ca. 4 mg) was added as internal standard. Then the NMR proton data were collected immediately. 

### 3.3. Synthesis of Metal Complexes

#### 3.3.1. (**L**)MnCl_2_

According to ref [56] MnCl_2_,4H_2_O (0.48 g, 2.4 mmol) was added to a solution of **L** (0.54 g, 2 mmol) in 3 mL of acetonitrile. The mixture was stirred at room temperature for 15 h and the solvent was removed under vacuum. The grey powder obtained was washed twice with diethyl ether and after recrystallization by diffusion of diethyl ether into a solution of the product in an acetonitrile-ethanol mixture, (**L**)MnCl_2_ (0.52 g, 65% yield) was obtained as a white powder.

Anal. Calc. for C_16_H_22_Cl_2_MnN_4_·0.5EtOH: C, 48.70; H, 6.01; N, 13.36. Found: C, 49.02; H, 5.98; N, 13.40.

#### 3.3.2. (**L**)Mn(OTf)_2_

According to ref [29], Mn(OTf)_2_ (0.875 g, 2.4 mmol) was added to a solution of **L** (0.54 g, 2 mmol) in 3 mL of acetonitrile. The mixture was stirred at room temperature for 15 h and the solvent was removed under vacuum. The light grey powder obtained was washed twice with diethyl ether and after recrystallization by diffusion of diethyl ether into a solution of the product in acetonitrile, (**L**)Mn(OTf)_2_ (0.85 g, 68% yield) was obtained as a white powder.

Anal. Calc. for C_18_H_22_F_6_MnN_4_O_6_S_2_: C, 34.68; H, 3.56; N, 8.99. Found: C, 34.68; H, 3.42; N, 8.95.

#### 3.3.3. (**L**)Mn(*p*-Ts)_2_

A solution of Ag(*p*-Ts) (1.34 g, 4.8 mmol) in 5 mL of H_2_O was added to a solution of (**L**)MnCl_2_ (0.79 g, 2 mmol) in 5 mL of H_2_O and the mixture was stirred at room temperature for 15 h. After removal of the AgCl precipitate by filtration, the solvent was removed under vacuum. Recrystallization of the crude product in absolute ethanol afforded (**L**)Mn(*p*-Ts)_2_ (0.96 g, 72% yield) as a grey solid.

Anal. Calc. for C_30_H_36_MnN_4_O_6_S_2_: C, 53.97; H, 5.43; N, 8.39. Found: C, 53.82; H, 5.50; N, 8.36.

#### 3.3.4. [(**L**)FeCl_2_](FeCl_4_)

FeCl_3_,6H_2_O (1.08 g, 4 mmol) was added to a solution of L (0.54 g, 2 mmol) in 5 mL of acetonitrile. After 15 min, a red precipitate appeared and the mixture was stirred for 15 h at room temperature. After filtration of the red solid recrystallization in CH_3_CN afforded [(**L**)FeCl_2_](FeCl_4_) (0.93 g, 73% yield) as a red solid. 

Anal. Calc. for C_16_H_22_Cl_6_Fe_2_N_4_: C, 32.31; H, 3.73; N, 9.42. Found: C, 32.39; H, 3.16; N, 9.33.

### 3.4. Synthesis of Silica Particles

#### 3.4.1. SiO_2_ Particles in EtOH (**SiO_2_**(E))

According to ref [64], 72 mL (4 mol) of H_2_O, 60 mL of ammonic solution (28% wt) were mixed in 630 mL (10.79 mol) of absolute ethanol at room temperature. A measure of 40 mL (0.18 mol) of tetraethylorthosilicate (TEOS) was added to the solution. A white suspension appeared. The mixture was stirred at 50 °C for 6 h. Then the solid was washed with absolute ethanol 5 times and collected by centrifugation. SiO_2_(E) particles were dried under vacuum at 120 °C overnight. A white powder was obtained. 

**SiO_2_(E)**: ^1^H NMR (400 MHz, D_2_O/NaOH-Benzoic acid) δ 7.57 (m, 2H, Ar-H), 7.21 (m, 3H, Ar-H), 3.31 (q, *J* = 7.1 Hz, 0.3H, CH_2_), 0.86 (t, *J* = 7.1 Hz,.0.43H, CH_3_). Anal. Found: C, 1.09; H, 0.67. ^29^Si CP MAS-NMR: -93.3 ppm (Q_2_), −101.9 ppm (Q_3_), −111.8 ppm (Q_4_). ^13^C CP MAS-NMR: 58.0 ppm (CH_2_O), 16.9 ppm (CH_3_). IR (ATR, ν(cm^−1^)): 3710-2935 (OH), 1059 (Si-O-Si), 949 (Si-OH), 790 and 438 (Si-O-Si).

#### 3.4.2. **SiO_2_@CN**(E) Particles

According to ref [68], a measure of 10 g of **SiO_2_(E)** particles was mixed with 25 mL of TESPN (0.11 mol) in 150 mL of toluene under stirring at 110°C for 6 days. The powder was washed 5 times with toluene, collected by centrifugation and dried under vacuum at 120°C overnight to obtain **SiO_2_@CN**(E) as a white powder.

^1^H NMR (400 MHz, D_2_O/NaOH-Benzoic acid) δ 7.66 (m, 2H, Ar-H), 7.29 (m, 3H, Ar-H), 3.42 (q, *J* = 7.1 Hz, 0.36H, CH_2_), 2.15 (m, 0.23H, CH_2_), 0.96 (t, *J* = 7.1 Hz, 0.54H, CH_3_), 0.54 (m, 0.24H, CH_2_). ^29^Si CP MAS-NMR: −62.2 ppm (T_2_), −70.4 ppm (T_3_), −92.8 ppm (Q_2_), −101.9 ppm (Q_3_), −111.9 ppm (Q_4_). ^13^C CP MAS-NMR: 120.9 ppm (CN), 60.2 ppm (CH_2_O), 58.1 ppm (CH_2_O), 16.4 ppm (CH_3_), 10.6 ppm (CH_2_Si), 8.8 ppm (CH_2_Si). IR (ATR, ν(cm^−1^)): 3712–2937 (OH), 2248 (CN), 1073 (Si-O-Si), 943 (Si-OH), 795 and 442 (Si-O-Si). ρ(CN) = 0.29 mmol/g. μ(CN) = 20.6 functions/nm^2^

#### 3.4.3. **SiO_2_@COOH**(E) Particles

According to ref [39], a measure of 5 g of **SiO_2_@CN**(E) was added to 50 mL of H_2_SO_4_ (65% wt, 0.52 mol) and the solution was heated at 150 °C under stirring for 4 h. A grey powder was found in suspension. Then the powder was washed with H_2_O until pH = 7. The product was collected by centrifugation and was dried under vacuum at 120°C. A light grey powder of **SiO_2_@COOH**(E) was obtained.

^1^H NMR (400 MHz, D_2_O/NaOH-Benzoic acid) δ 7.58 (m, 2H, Ar-H), 7.21 (m, 3H, Ar-H), 3.33 (q, *J* = 7.1 Hz, 0.16H, CH_2_), 1.91 (m, 0.02H, CH_2_), 0.87 (t, *J* = 7.1 Hz, 0.23H, CH_3_), 0.54 (m, 0.03H, CH_2_). ^29^Si CP MAS-NMR: -59.6 ppm (T_2_), −68.7 ppm (T_3_), −92.8 ppm (Q_2_), −101.9 ppm (Q_3_), −111.7 ppm (Q_4_).^13^C CP MAS-NMR: 60.0 ppm (CH_2_O), 58.8 ppm (CH_2_O), 16.6 ppm (CH_3_). IR (ATR, ν(cm^−1^)): 3709–2933 (OH), 1737–1716 (C=O), 1073 (Si-O-Si), 943 (Si-OH), 794 and 446 (Si-O-Si). ρ(COOH) = 0.04 mmol/g. μ(COOH) = 2.8 functions/nm^2^.

#### 3.4.4. SiO_2_ Nanoparticles in Methanol (**SiO_2_**(M)) 

A measure of 72 mL (4 mol) of H_2_O and 60 mL of ammonic solution (28 wt.%) were mixed in 630 mL (15.57 mol) of methanol at room temperature. A measure of 40 mL (0.18 mol) of tetraethyl orthosilicate (TEOS) was added into the solution. A suspension of a white solid appeared. The mixture was stirred at 50 °C for 6 h. The solid was washed with absolute ethanol 5 times, collected by centrifugation and dried under vacuum at 120°C overnight. A white powder of **SiO_2_(M)** was obtained.

^1^H NMR (400 MHz, D_2_O/NaOH-Benzoic acid) δ 7.62 (m, 2H, Ar-H), 7.25 (m, 3H, Ar-H), 3.06 (s, 0.04H, CH_3_). ^29^Si CP MAS-NMR: −93.3 ppm (Q_2_), −101.9 ppm (Q_3_), −111.7 ppm (Q_4_). ^13^C CP MAS-NMR: 58.2 ppm (CH_2_O), 16.7 ppm (CH_3_). IR (ATR, ν(cm^−1^)): 3732–2850 (OH), 1062 (Si-O-Si), 945 (Si-OH), 784 and 443 (Si-O-Si). 

#### 3.4.5. **SiO_2_@CN**(M) Nanoparticles

A measure of 10 g of SiO_2_(M) was mixed with 25 mL of TESPN (0.11 mol) in 150 mL of toluene at 110°C under stirring for 6 days. The solid was washed 5 times with toluene, collected by centrifugation and dried under vacuum at 120 °C overnight. A white powder of **SiO_2_@CN**(M) was obtained. 

^1^H NMR (400 MHz, D_2_O/NaOH-Benzoic acid) δ 7.66 (m, 2H, Ar-H), 7.30 (m, 3H, Ar-H), 3.43 (q, *J* = 7.1 Hz, 3.16H, CH_2_), 3.12 (s, 0.06H, CH_3_), 2.20 (m, 1.98H, CH_2_), 0.96 (t, *J* = 7.1 Hz, 4.76H, CH_3_) 0.54 (m, 2.02H, CH_2_). ^29^Si CP MAS-NMR: −64.7 ppm (T_2_), −70.5 ppm (T_3_), −93.3 ppm (Q_2_), −102.4 ppm (Q_3_), −111.7 ppm (Q_4_). ^13^C CP MAS-NMR: 121.0 ppm (CN), 59.9 ppm (CH_2_O), 16.8 ppm (CH_3_), 10.5 ppm (CH_2_Si), 8.8 ppm (CH_2_Si). IR (ATR, ν(cm^−1^)): 3721-2903 (OH), 2253 (CN), 1065 (Si-O-Si), 941 (Si-OH), 788 and 425 (Si-O-Si). ρ(CN) = 1.40 mmol/g. μ(CN) = 16.6 functions/nm^2^

#### 3.4.6. **SiO_2_@COOH**(M) Nanoparticles

A measure of 5 g of **SiO_2_@CN**(M) was added to 50 mL of H_2_SO_4_ (65% wt, 0.52 mol) and the solution was heated at 150 °C under stirring for 4 h. A grey powder was found in suspension. The powder was washed with H_2_O until pH = 7. The product was collected by centrifugation and was dried under vacuum at 120 °C. A light grey powder of **SiO_2_@COOH**(M) was obtained.

^1^H NMR (400 MHz, D_2_O/NaOH-Benzoic acid) δ 7.66 (m, 2H, Ar-H), 7.29 (m, 3H, Ar-H), 3.42 (q, *J* = 7.1 Hz, 0.03H, CH_2_), 3.12 (s, 0.03H, CH_3_), 1.99 (m, 0.12H, CH_2_), 1.02 (t, *J* = 7.1 Hz, 0.04H, CH_3_), 0.46 (m, 0.13H, CH_2_). ^29^Si CP MAS-NMR: −58.8 ppm (T_2_), −68.4 ppm (T_3_), −91.9 ppm (Q_2_), −101.8 ppm (Q_3_), −111.6 ppm (Q_4_). ^13^C CP MAS-NMR: 177.9 ppm (COOH), 59.9 ppm (CH_2_O), 49.5 ppm (CH_2_O), 16.7 ppm (CH_3_), 6.7 ppm (CH_2_Si).IR (ATR, ν(cm^−1^)): 3709–2852 (OH), 1717 (C=O), 1046 (Si-O-Si), 932 (Si-OH), 785 and 450 (Si-O-Si). ρ(COOH) = 0.31 mmol/g. μ(COOH) = 3.2 functions/nm^2^.

### 3.5. Catalytic Experiments

#### 3.5.1. General Procedure of Catalysis with CH_3_COOH

A measure of 1 mmol of substrate (CO, CH. CYol), 0.84 g (14 mmol or 0.14 mmol) of CH_3_COOH, 0.01 mmol of complexes ((**L**)MnCl_2_, (**L**)Mn(OTf)_2_, (**L**)Mn(*p*-Ts)_2_, [(**L**)FeCl_2_](FeCl_4_)) and some drops of an internal standard (acetophenone) were mixed in 2 mL of CH_3_CN at room temperature. A measure of 0.13 mL of H_2_O_2_ (35 wt.% in H_2_O) diluted into 0.87 mL of CH_3_CN was slowly added into the mixture for 2 h at 0 °C. The mixture was left for 1 h at 0 °C.

#### 3.5.2. General Procedure of Catalysis with **SiO_2_@COOH**

A measure of 1 mmol of substrate (CO, CH, CYol), 300 mg of **SiO_2_@COOH**(E) (13.5 mg for **SiO_2_@COOH**(M) (0.14 mmol of carboxylic function), 0.01 mmol of complexes ((**L**)MnCl_2_, (**L**)Mn(OTf)_2_, (**L**)Mn(*p*-Ts)_2_, [(**L**)FeCl_2_](FeCl_4_)) and some drops of an internal standard (acetophenone) were mixed in 2 mL of CH_3_CN at room temperature. A measure of 0.13 mL of H_2_O_2_ (35 wt.% in H_2_O) diluted in 0.87 mL of CH_3_CN was slowly added to the mixture for 3 h at 50 °C. Then the mixture was left at 60 °C for 2 h.

## 4. Conclusions

It has been possible to replace acetic acid with silica beads with carboxylic functions in the reaction of the epoxidation of olefins. The study showed lower activity with the silica beads in the case of cyclooctene and cyclohexene oxidation with manganese complexes and selectivity seemed to be linked to the nature of the ion of the complex. With cyclohexene, the activity with the beads was higher relatively to cyclooctene. However, for the Fe complex, the beads were more active than acetic acid. With cyclohexanol, the process worked much better with acetic acid. The size of the bead seemed to have no relevant effect in terms of efficiency, except that the quantity of carboxylic functions brought into the reaction was 100 times less than the quantity of acetic acid. It should be noted that under a lower quantity of acetic acid, the reaction did not work. Although less active, this method is the first step towards the replacement of an organic volatile reagent.

## Figures and Tables

**Table 1:** Selected bond distances (Å) and angles (deg.) for (**L**)Mn(*p*-Ts)_2_ and [(**L**)FeCl_2_](FeCl_4_).

	(L)Mn(*p*-Ts)_2_	[(L)FeCl_2_](FeCl_4_)
Bonds (Å)		
M-N_py_	2.308(5)–2.352(2)	2.1408(12), 2.1556(12)
M-N_amine_	2.249(2)–2.283(2)	2.2233(11), 2.2264(12)
Angles (°)		
N_amine_-M-N_amine_	75.46(9)–76.06(8)	79.70(4)
N_Py_-M-N_Py_	168.55(8)–168.87(7)	166.17(5)

**Table 2:** Number of functions (F) (mmol) per g sample, calculated by ^1^H NMR.

S	ρ(f) (mmol F/g S)			
	OCH_2_CH_3_	OCH_3_	CN	COOH
**SiO_2_** (E)	0.43			
**SiO_2_@CN** (E)	0.64		0.29	
**SiO_2_@COOH** (E)	0.45			0.04
**SiO_2_** (M)	1.18	0.05		
**SiO_2_@CN** (M)	1.85	0.04	1.40	
**SiO_2_@COOH** (M)	0.08	0.05		0.31

**Table 3:** Number of function (mol) per nm^2^ core (μ(f)).

Solvent Used for SiO_2_ Synthesis	SiO_2_@CN	SiO_2_@COOH
Ethanol	20.6	2.8
Methanol	16.6	3.2

**Table 4:** Relevant data for the catalyzed epoxidation of CO.^(a).^

Catalyst	RCOOH	CO	COE		TON ^(e^^)^
		Conv ^(b^^)^	Sel ^(c^^)^	Yield ^(d^^)^	
(**L**)MnCl_2_	no	1	-	-	-
	CH_3_COOH	99	81	81	100
	CH_3_COOH ^(^^f)^	1	-	-	-
	**SiO_2_@COOH**(M)	37	9	4	38
	**SiO_2_@COOH**(E)	55	26	14	55
(**L**)Mn(OTf)_2_	no	5	7	<1	3
	CH_3_COOH	99	54	54	99
	**SiO_2_@COOH**(M)	50	45	23	50
	**SiO_2_@COOH**(E)	53	43	23	52
(**L**)Mn(*p*-Ts)_2_	no	5	50	2.7	6
	CH_3_COOH	100	62	62	100
	**SiO_2_@COOH**(M)	61	30	19	61
	**SiO_2_@COOH**(E)	62	28	23	62
[(**L**)FeCl_2_](FeCl_4_)	no	0	-	-	-
	CH_3_COOH	60	21	13	60
	**SiO_2_@COOH**(M)	80	31	25	80
	**SiO_2_@COOH**(E)	91	25	23	91

^(a)^ Experimental conditions: 0 °C with CH_3_COOH, 60 °C with **SiO_2_@COOH**. Cat/H_2_O_2_/CO/CH_3_COOH = 1/150/100/1400 for CH_3_COOH, t = 3 h; Cat/H_2_O_2_/CO/COOH = 1/150/100/14 for **SiO_2_@COOH**, t = 5 h. ^(b)^ nCO converted/nCO engaged (%) at the end of the reaction. ^(c)^ nCOE formed/nCO converted at the end of the reaction. ^(d)^ nCOE formed/nCO engaged at the end of the reaction. ^(e)^ nCO transformed/ncat at the end of the reaction. ^(f)^ Cat/H_2_O_2_/CO/CH_3_COOH=1/150/100/14, t = 3 h, 0 °C.

**Table 5:** Relevant data for the catalyzed (ep)oxidation of cyclohexene ^(a)^.

Catalyst	RCOOH	Conv ^(b)^	Selectivity ^(c)^				TON ^(d)^
		CH	CHO	CHD	CHol	CHone	
(**L**)MnCl_2_	CH_3_COOH	100	89	0	0	0	100
	**SiO_2_@COOH**(M)	63	3.3	0	2	2	63
	**SiO_2_@COOH**(E)	74	14	23	0	0	74
(**L**)Mn(OTf)_2_	CH_3_COOH	98	57	3	0	1	98
	**SiO_2_@COOH**(M)	57	13	0	0	0	56
	**SiO_2_@COOH**(E)	83	27	0	0	0	83
(**L**)Mn(*p*-Ts)_2_	CH_3_COOH	96	68	2	0	2	96
	**SiO_2_@COOH**(M)	53	16	0	0	0	53
	**SiO_2_@COOH**(E)	80	28	0	0	0	80
[(**L**)FeCl_2_](FeCl_4_)	CH_3_COOH	11	0	0	0	0	11
	**SiO_2_@COOH**(M)	87	9	23	6	17	86
	**SiO_2_@COOH**(E)	96	4	5	0	9	96

^(a)^ Conditions: 0 °C for the case with CH_3_COOH, 60 °C for the case with **SiO_2_@COOH**. Cat/H_2_O_2_/CH/CH_3_COOH = 1/150/100/1400 for CH_3_COOH, t = 3 h; Cat/H_2_O_2_/CH/COOH = 1/150/100/14 for **SiO_2_@COOH**, t = 5 h. ^(b)^ n_CH_ converted/n_CH_ engaged (in%) after 3 h for CH_3_COOH, 5 h for **SiO_2_@COOH**. ^(c)^ n_product_ formed/ n_CH_ converted at 3 h for CH_3_COOH, 5 h for **SiO_2_@COOH**. ^(d)^ n_CH_ transformed /n_Cat_ at 3 h for CH_3_COOH, 5 h for **SiO_2_@COOH**.

**Table 6:** Relevant data for the catalyzed oxidation of cyclohexanol ^(a)^.

Catalyst	RCOOH	CYol	CYone		TON ^(e)^
		Conv ^(b)^	Sel ^(c)^	Yield ^(d)^	
(**L**)MnCl_2_	CH_3_COOH	81	91	74	81
	**SiO_2_@COOH**(M)	15	46	7	15
	**SiO_2_@COOH**(E)	16	90	14	16
(**L**)Mn(OTf)_2_	CH_3_COOH	100	79	79	100
	**SiO_2_@COOH**(M)	23	90	21	23
	**SiO_2_@COOH**(E)	27	87	24	27
(**L**)Mn(*p*-Ts)_2_	CH_3_COOH	99	85	85	99
	**SiO_2_@COOH**(M)	21	97	21	21
	**SiO_2_@COOH**(E)	25	87	22	25
[(**L**)FeCl_2_](FeCl_4_)	CH_3_COOH	100	79	79	99
	**SiO_2_@COOH**(M)	59	45	27	59
	**SiO_2_@COOH**(E)	65	36	23	65

^(a)^ Conditions: 0 °C for the case with CH_3_COOH, 60 °C for the case with **SiO_2_@COOH** Cat/H_2_O_2_/CYol/CH_3_COOH = 1/150/100/1400 for CH_3_COOH, t = 3 h; Cat/H_2_O_2_/CYol/COOH = 1/150/100/14 for **SiO_2_@COOH**, t = 5 h. ^(b)^ n_CYol_ converted/n_CYol_ engaged (in%) after 3 h for CH_3_COOH, 5 h for **SiO_2_@COOH**. ^(c)^ n_CYone_ formed/ n_CYol_ converted at 3 h for CH_3_COOH, 5h for **SiO_2_@COOH**. ^(d)^ n_CYone_ formed/ n_CYol_ engaged at 3h for CH_3_COOH, 5 h for **SiO_2_@COOH**. ^(e)^ n_CYol_ transformed /n_Cat_ at 3 h for CH_3_COOH, 5 h for **SiO_2_@COOH**.

## Data Availability

Data is contained within the article or Supplementary Material.

## Supplementary Materials

The following are available online, Table S1: Crystal data. Table S2: Bond lengths [Å] and angles [°] for (**L**)Mn(*p*-Ts)_2_. Table S3: Bond lengths [Å] and angles [°] for [(**L**)FeCl_2_](FeCl_4_). Table S4: Relevant solid-state NMR data. Table S5: ^1^H NMR chemical shifts (in ppm) observed with **SiO_2_**, **SiO_2_@CN** and **SiO_2_@COOH** in D_2_O/NaOH (pH=13) solution. Figure S1: ^13^C MAS NMR spectra of **SiO_2_** (bottom), **SiO_2_@CN** (middle) and **SiO_2_@COOH** (top) for beads from **SiO_2_** beads produced in EtOH (left) and MeOH (right). Figure S2: ^29^Si MAS NMR spectra of **SiO_2_** (top) **SiO_2_@CN** (middle), **SiO_2_@COOH** (bottom) from **SiO_2_** beads produced in EtOH (left) and MeOH (right).

## Sample Availability

Samples of the compounds are available from the authors.
