# Supplementary material for: Replacement of Volatile Acetic Acid by Solid SiO2@COOH Silica (Nano)Beads for (Ep)Oxidation Using Mn and Fe Complexes Containing BPMEN Ligand
Source: Molecules. 2021 Sep 7;26(18):5435. doi: 10.3390/molecules26185435 (PMC8470966; doi:10.3390/molecules26185435)
Supplement: Supplementary file 1 [file molecules-26-05435-s001.zip › molecules-1322801-supplementary.pdf]

Replacement of volatile acetic acid by solid SiO_2@COOH silica (nano)beads for (ep)oxidation using Mn and Fe complexes containing BPMEN ligand

Yun Wang, Florence Gayet, Jean-Claude Daran, Pascal Guillo* and Dominique Agustin*

Supplementary Information

Table S1 –Crystal data.

Identification code	(**L**)Mn(*p*-Ts)$_2$	[(**L**)$FeCl_2$]($FeCl_4$)
Empirical formula	$C_{60}H_{72}Mn_2N_8O_{12}S_4$	$C_{16}H_{22}Cl_6Fe_2N_4$
Formula weight	1335.37	594.77
Temperature, K	173(2)	173(2)
Wavelength, Å	0.71073	0.71073
Crystal system	Monoclinic	Monoclinic
Space group	P 21/c	P 21/n
a, Å	18.087(9)	8.3247(3)
b, Å	18.486(9)	23.9715(10)
c, Å	19.201(11)	12.1723(5)
α,°	90	90
β, °	101.428(11)	98.6680(10)
γ,°	90	90
Volume, Å^3	6293(5)	2401.31(17)
Z	4	4
Density (calc), Mg/m^3	1.410	1.645
Abs. coefficient, mm^{-1}	0.601	1.886
F(000)	2792	1200
Crystal size, mm^3	0.500 x 0.400 x 0.250	0.500 x 0.430 x 0.100
Theta range, °	1.149 to 26.372	2.907 to 36.553
Reflections collected	126193	108690
Indpt reflections (R_{int})	12854 (0.1852	11715 (0.0357)
Completeness, %	99.6	99.9
Absorption correction	Semi-empirical from equivalents	Semi-empirical from equivalents
Refinement method	Full-matrix least-squares on F^2	Full-matrix least-squares on F^2
Data /restraints/parameters	12854 / 7 / 810	11715 / 0 / 255
Goodness-of-fit on F^2	1.047	1.094
R1, wR2 [I>2σ(I)]	0.0483, 0.1284	0.0387, 0.0789
R1, wR2 (all data)	0.0675, 0.1431	0.0552, 0.0875
Residual density, e.Å^{-3}	0.535 / -1.082	1.013 and -0.807

Table S2 - Bond lengths [Å] and angles [°] for (**L**)Mn(*p*-Ts)$_2$.

Mn(1)-O(21)	2.057(2)	Mn(2)-O(31B)	2.152(4)
Mn(1)-O(11)	2.1078(19)	Mn(2)-N(21)	2.271(2)
Mn(1)-N(12)	2.249(2)	Mn(2)-N(22)	2.276(2)
Mn(1)-N(11)	2.283(2)	Mn(2)-N(24)	2.325(2)
Mn(1)-N(14)	2.308(2)	Mn(2)-N(23)	2.326(2)
Mn(1)-N(13)	2.352(2)	O(31A)-S(3)	1.439(3)
S(1)-O(13)	1.3984(19)	S(3)-O(32A)	1.392(3)
S(1)-O(12)	1.427(2)	S(3)-O(31B)	1.406(4)
S(1)-O(11)	1.4663(19)	S(3)-O(33A)	1.430(3)
S(1)-C(11)	1.850(2)	S(3)-O(33B)	1.452(4)
S(2)-O(21)	1.401(2)	S(3)-O(32B)	1.474(4)
S(2)-O(23)	1.402(2)	S(3)-C(31)	1.852(2)
S(2)-O(22)	1.473(2)	S(4)-O(43)	1.385(3)
S(2)-C(21)	1.850(3)	S(4)-O(41)	1.388(2)
N(11)-C(111)	1.439(4)	S(4)-O(42)	1.437(3)
N(11)-C(113)	1.474(3)	S(4)-C(41)	1.856(3)
N(11)-C(112)	1.561(4)	N(21)-C(211)	1.451(3)
N(12)-C(121)	1.474(3)	N(21)-C(213)	1.485(3)
N(12)-C(123)	1.482(3)	N(21)-C(212)	1.562(4)
N(12)-C(122)	1.542(4)	N(22)-C(221)	1.430(3)
N(13)-C(124)	1.282(3)	N(22)-C(223)	1.475(3)
N(13)-C(128)	1.400(3)	N(22)-C(222)	1.567(4)
N(14)-C(114)	1.292(3)	N(23)-C(224)	1.294(3)
N(14)-C(118)	1.400(4)	N(23)-C(228)	1.408(3)
C(11)-C(16)	1.317(3)	N(24)-C(214)	1.285(3)
C(11)-C(12)	1.413(3)	N(24)-C(218)	1.404(4)
C(12)-C(13)	1.452(4)	C(31)-C(36)	1.318(3)
C(13)-C(14)	1.320(3)	C(31)-C(32)	1.405(4)
C(14)-C(15)	1.413(4)	C(32)-C(33)	1.441(4)
C(14)-C(17)	1.578(4)	C(33)-C(34)	1.320(4)
C(15)-C(16)	1.459(4)	C(34)-C(35)	1.412(4)
C(21)-C(22)	1.352(3)	C(34)-C(37)	1.578(4)
C(21)-C(26)	1.376(4)	C(35)-C(36)	1.452(4)
C(22)-C(23)	1.443(4)	C(41)-C(42)	1.338(3)
C(23)-C(24)	1.374(5)	C(41)-C(46)	1.400(3)
C(24)-C(25)	1.352(4)	C(42)-C(43)	1.443(4)
C(24)-C(27)	1.580(4)	C(43)-C(44)	1.392(5)
C(25)-C(26)	1.448(4)	C(44)-C(45)	1.340(4)
C(111)-C(114)	1.573(5)	C(44)-C(47)	1.574(4)
C(113)-C(123)	1.444(4)	C(45)-C(46)	1.452(4)
C(114)-C(115)	1.402(4)	C(211)-C(214)	1.583(4)
C(115)-C(116)	1.438(5)	C(213)-C(223)	1.433(4)
C(116)-C(117)	1.332(5)	C(214)-C(215)	1.418(4)
C(117)-C(118)	1.386(4)	C(215)-C(216)	1.451(5)
C(121)-C(124)	1.567(4)	C(216)-C(217)	1.317(5)
C(124)-C(125)	1.431(4)	C(217)-C(218)	1.412(4)
C(125)-C(126)	1.434(5)	C(221)-C(224)	1.589(4)
C(126)-C(127)	1.316(5)	C(224)-C(225)	1.403(4)
C(127)-C(128)	1.424(4)	C(225)-C(226)	1.448(4)
Mn(2)-O(31A)	2.083(3)	C(226)-C(227)	1.322(4)
Mn(2)-O(41)	2.094(2)	C(227)-C(228)	1.411(4)

O(21)-Mn(1)-O(11)	101.49(9)	O(41)-Mn(2)-N(22)	92.80(10)
O(21)-Mn(1)-N(12)	158.57(8)	O(31B)-Mn(2)-N(22)	153.72(13)
O(11)-Mn(1)-N(12)	87.58(8)	N(21)-Mn(2)-N(22)	75.46(8)
O(21)-Mn(1)-N(11)	100.59(9)	O(31A)-Mn(2)-N(24)	102.93(11)
O(11)-Mn(1)-N(11)	154.73(8)	O(41)-Mn(2)-N(24)	81.90(8)
N(12)-Mn(1)-N(11)	76.06(8)	O(31B)-Mn(2)-N(24)	96.50(13)
O(21)-Mn(1)-N(14)	97.80(8)	N(21)-Mn(2)-N(24)	77.82(8)
O(11)-Mn(1)-N(14)	87.56(8)	N(22)-Mn(2)-N(24)	94.26(8)
N(12)-Mn(1)-N(14)	101.98(8)	O(31A)-Mn(2)-N(23)	86.28(11)
N(11)-Mn(1)-N(14)	77.40(8)	O(41)-Mn(2)-N(23)	104.63(8)
O(21)-Mn(1)-N(13)	81.96(8)	O(31B)-Mn(2)-N(23)	89.37(13)
O(11)-Mn(1)-N(13)	103.73(8)	N(21)-Mn(2)-N(23)	93.53(8)
N(12)-Mn(1)-N(13)	77.01(8)	N(22)-Mn(2)-N(23)	76.62(7)
N(11)-Mn(1)-N(13)	91.36(8)	N(24)-Mn(2)-N(23)	168.87(7)
N(14)-Mn(1)-N(13)	168.55(8)	S(3)-O(31A)-Mn(2)	148.6(3)
O(13)-S(1)-O(12)	109.94(13)	O(32A)-S(3)-O(33A)	116.7(3)
O(13)-S(1)-O(11)	115.35(12)	O(32A)-S(3)-O(31A)	117.0(2)
O(12)-S(1)-O(11)	110.04(12)	O(33A)-S(3)-O(31A)	105.7(3)
O(13)-S(1)-C(11)	106.64(11)	O(31B)-S(3)-O(33B)	111.8(3)
O(12)-S(1)-C(11)	112.49(11)	O(31B)-S(3)-O(32B)	112.5(3)
O(11)-S(1)-C(11)	102.18(10)	O(33B)-S(3)-O(32B)	103.6(3)
O(21)-S(2)-O(23)	108.89(16)	O(32A)-S(3)-C(31)	110.38(18)
O(21)-S(2)-O(22)	110.78(13)	O(31B)-S(3)-C(31)	107.55(19)
O(23)-S(2)-O(22)	114.98(17)	O(33A)-S(3)-C(31)	105.00(17)
O(21)-S(2)-C(21)	102.22(12)	O(31A)-S(3)-C(31)	100.19(17)
O(23)-S(2)-C(21)	109.11(13)	O(33B)-S(3)-C(31)	114.09(19)
O(22)-S(2)-C(21)	110.09(12)	O(32B)-S(3)-C(31)	107.21(19)
S(1)-O(11)-Mn(1)	148.08(13)	S(3)-O(31B)-Mn(2)	144.4(3)
S(2)-O(21)-Mn(1)	152.19(14)	O(43)-S(4)-O(41)	108.0(2)
C(111)-N(11)-C(113)	106.3(2)	O(43)-S(4)-O(42)	112.2(3)
C(111)-N(11)-C(112)	113.0(2)	O(41)-S(4)-O(42)	113.37(17)
C(113)-N(11)-C(112)	111.7(2)	O(43)-S(4)-C(41)	111.37(15)
C(111)-N(11)-Mn(1)	104.68(17)	O(41)-S(4)-C(41)	103.99(12)
C(113)-N(11)-Mn(1)	106.54(16)	O(42)-S(4)-C(41)	107.64(13)
C(112)-N(11)-Mn(1)	113.99(17)	S(4)-O(41)-Mn(2)	149.56(16)
C(121)-N(12)-C(123)	107.9(2)	C(211)-N(21)-C(213)	107.9(2)
C(121)-N(12)-C(122)	114.9(2)	C(211)-N(21)-C(212)	114.1(2)
C(123)-N(12)-C(122)	110.2(2)	C(213)-N(21)-C(212)	110.9(2)
C(121)-N(12)-Mn(1)	101.94(15)	C(211)-N(21)-Mn(2)	101.62(16)
C(123)-N(12)-Mn(1)	109.15(16)	C(213)-N(21)-Mn(2)	108.37(16)
C(122)-N(12)-Mn(1)	112.33(16)	C(212)-N(21)-Mn(2)	113.40(16)
C(124)-N(13)-C(128)	116.0(2)	C(221)-N(22)-C(223)	106.2(2)
C(124)-N(13)-Mn(1)	113.29(18)	C(221)-N(22)-C(222)	113.2(2)
C(128)-N(13)-Mn(1)	130.68(17)	C(223)-N(22)-C(222)	112.1(2)
C(114)-N(14)-C(118)	118.7(2)	C(221)-N(22)-Mn(2)	103.27(15)
C(114)-N(14)-Mn(1)	112.26(19)	C(223)-N(22)-Mn(2)	108.97(15)
C(118)-N(14)-Mn(1)	127.94(17)	C(222)-N(22)-Mn(2)	112.46(16)
C(16)-C(11)-C(12)	116.0(2)	C(224)-N(23)-C(228)	118.1(2)
C(16)-C(11)-S(1)	119.56(19)	C(224)-N(23)-Mn(2)	112.04(17)
C(12)-C(11)-S(1)	124.42(17)	C(228)-N(23)-Mn(2)	129.74(16)
C(11)-C(12)-C(13)	124.6(2)	C(214)-N(24)-C(218)	117.4(2)
C(14)-C(13)-C(12)	120.5(2)	C(214)-N(24)-Mn(2)	112.36(18)
C(13)-C(14)-C(15)	113.9(2)	C(218)-N(24)-Mn(2)	130.13(17)
C(13)-C(14)-C(17)	119.5(3)	C(36)-C(31)-C(32)	115.9(2)
C(15)-C(14)-C(17)	126.6(2)	C(36)-C(31)-S(3)	117.87(18)
C(14)-C(15)-C(16)	126.5(2)	C(32)-C(31)-S(3)	126.27(18)

C(11)-C(16)-C(15)	118.4(2)	C(31)-C(32)-C(33)	124.8(2)
C(22)-C(21)-C(26)	115.9(2)	C(34)-C(33)-C(32)	120.6(2)
C(22)-C(21)-S(2)	121.6(2)	C(33)-C(34)-C(35)	114.1(2)
C(26)-C(21)-S(2)	122.53(18)	C(33)-C(34)-C(37)	120.1(2)
C(21)-C(22)-C(23)	120.5(3)	C(35)-C(34)-C(37)	125.8(2)
C(24)-C(23)-C(22)	124.7(3)	C(34)-C(35)-C(36)	125.9(2)
C(25)-C(24)-C(23)	114.1(3)	C(31)-C(36)-C(35)	118.8(2)
C(25)-C(24)-C(27)	121.3(3)	C(42)-C(41)-C(46)	116.3(2)
C(23)-C(24)-C(27)	124.5(3)	C(42)-C(41)-S(4)	120.8(2)
C(24)-C(25)-C(26)	122.2(3)	C(46)-C(41)-S(4)	122.83(19)
C(21)-C(26)-C(25)	122.6(2)	C(41)-C(42)-C(43)	119.8(3)
N(11)-C(111)-C(114)	117.3(2)	C(44)-C(43)-C(42)	125.1(3)
C(123)-C(113)-N(11)	107.7(2)	C(45)-C(44)-C(43)	114.5(3)
N(14)-C(114)-C(115)	117.6(3)	C(45)-C(44)-C(47)	120.1(3)
N(14)-C(114)-C(111)	117.6(2)	C(43)-C(44)-C(47)	125.4(3)
C(115)-C(114)-C(111)	124.6(3)	C(44)-C(45)-C(46)	121.5(3)
C(114)-C(115)-C(116)	122.9(3)	C(41)-C(46)-C(45)	122.7(2)
C(117)-C(116)-C(115)	119.3(3)	N(21)-C(211)-C(214)	116.4(2)
C(116)-C(117)-C(118)	114.6(3)	C(223)-C(213)-N(21)	109.6(2)
C(117)-C(118)-N(14)	126.9(3)	N(24)-C(214)-C(215)	117.9(3)
N(12)-C(121)-C(124)	117.9(2)	N(24)-C(214)-C(211)	114.9(2)
C(113)-C(123)-N(12)	108.7(2)	C(215)-C(214)-C(211)	127.1(2)
N(13)-C(124)-C(125)	118.7(3)	C(214)-C(215)-C(216)	123.8(3)
N(13)-C(124)-C(121)	113.7(2)	C(217)-C(216)-C(215)	118.0(3)
C(125)-C(124)-C(121)	127.5(2)	C(216)-C(217)-C(218)	115.0(3)
C(124)-C(125)-C(126)	124.3(3)	N(24)-C(218)-C(217)	127.7(3)
C(127)-C(126)-C(125)	117.3(3)	N(22)-C(221)-C(224)	115.9(2)
C(126)-C(127)-C(128)	115.4(3)	C(213)-C(223)-N(22)	107.6(2)
N(13)-C(128)-C(127)	128.2(3)	N(23)-C(224)-C(225)	118.2(3)
O(31A)-Mn(2)-O(41)	89.78(14)	N(23)-C(224)-C(221)	115.9(2)
O(41)-Mn(2)-O(31B)	112.40(15)	C(225)-C(224)-C(221)	125.8(2)
O(31A)-Mn(2)-N(21)	107.81(13)	C(224)-C(225)-C(226)	123.3(2)
O(41)-Mn(2)-N(21)	155.60(9)	C(227)-C(226)-C(225)	118.7(2)
O(31B)-Mn(2)-N(21)	83.48(15)	C(226)-C(227)-C(228)	115.3(3)
O(31A)-Mn(2)-N(22)	162.81(11)	N(23)-C(228)-C(227)	126.3(2)

Table S3 - Bond lengths [Å] and angles [°] for [(**L**)$FeCl_2$]($FeCl_4$).

Fe(1)-N(2)	2.1408(12)	C(21)-C(32)	1.501(2)
Fe(1)-N(1)	2.1556(12)	C(22)-C(23)	1.383(2)
Fe(1)-N(4)	2.2233(11)	C(22)-H(22)	0.9500
Fe(1)-N(3)	2.2264(12)	C(23)-C(24)	1.385(3)
Fe(1)-Cl(2)	2.2697(4)	C(23)-H(23)	0.9500
Fe(1)-Cl(1)	2.2784(4)	C(24)-C(25)	1.383(2)
N(1)-C(11)	1.3463(19)	C(24)-H(24)	0.9500
N(1)-C(15)	1.3487(19)	C(25)-H(25)	0.9500
N(2)-C(21)	1.3480(18)	C(32)-H(32A)	0.9900
N(2)-C(25)	1.3502(19)	C(32)-H(32B)	0.9900
N(3)-C(32)	1.4831(19)	C(33)-H(33A)	0.9800
N(3)-C(33)	1.4842(19)	C(33)-H(33B)	0.9800
N(3)-C(34)	1.4945(18)	C(33)-H(33C)	0.9800
N(4)-C(42)	1.4768(19)	C(34)-C(44)	1.510(2)
N(4)-C(43)	1.4888(18)	C(34)-H(34A)	0.9900
N(4)-C(44)	1.4915(18)	C(34)-H(34B)	0.9900
C(11)-C(12)	1.387(2)	C(42)-H(42A)	0.9900
C(11)-C(42)	1.496(2)	C(42)-H(42B)	0.9900
C(12)-C(13)	1.382(3)	C(43)-H(43A)	0.9800
C(12)-H(12)	0.9500	C(43)-H(43B)	0.9800
C(13)-C(14)	1.382(3)	C(43)-H(43C)	0.9800
C(13)-H(13)	0.9500	C(44)-H(44A)	0.9900
C(14)-C(15)	1.381(2)	C(44)-H(44B)	0.9900
C(14)-H(14)	0.9500	Fe(2)-Cl(23)	2.1863(5)
C(15)-H(15)	0.9500	Fe(2)-Cl(21)	2.1948(5)
C(21)-C(22)	1.382(2)	Fe(2)-Cl(24)	2.1990(6)
		Fe(2)-Cl(22)	2.2034(5)

N(2)-Fe(1)-N(1)	166.17(5)	C(23)-C(24)-H(24)	120.5
N(2)-Fe(1)-N(4)	92.63(5)	N(2)-C(25)-C(24)	122.12(15)
N(1)-Fe(1)-N(4)	75.10(4)	N(2)-C(25)-H(25)	118.9
N(2)-Fe(1)-N(3)	76.28(5)	C(24)-C(25)-H(25)	118.9
N(1)-Fe(1)-N(3)	94.95(5)	N(3)-C(32)-C(21)	109.25(11)
N(4)-Fe(1)-N(3)	79.70(4)	N(3)-C(32)-H(32A)	109.8
N(2)-Fe(1)-Cl(2)	96.39(4)	C(21)-C(22)-H(22)	120.3
N(1)-Fe(1)-Cl(2)	94.22(3)	C(23)-C(22)-H(22)	120.3
N(4)-Fe(1)-Cl(2)	164.15(3)	C(13)-C(14)-H(14)	120.4
N(3)-Fe(1)-Cl(2)	89.80(3)	N(1)-C(15)-C(14)	122.05(16)
N(2)-Fe(1)-Cl(1)	93.43(4)	N(1)-C(15)-H(15)	119.0
N(1)-Fe(1)-Cl(1)	93.41(3)	C(14)-C(15)-H(15)	119.0
N(4)-Fe(1)-Cl(1)	92.30(3)	N(2)-C(21)-C(22)	121.72(14)
N(3)-Fe(1)-Cl(1)	166.52(3)	C(21)-C(32)-H(32A)	109.8
Cl(2)-Fe(1)-Cl(1)	100.112(16)	N(3)-C(32)-H(32B)	109.8
C(11)-N(1)-C(15)	118.59(13)	C(21)-C(32)-H(32B)	109.8
C(11)-N(1)-Fe(1)	114.33(9)	H(32A)-C(32)-H(32B)	108.3
C(15)-N(1)-Fe(1)	126.33(11)	N(3)-C(33)-H(33A)	109.5
C(21)-N(2)-C(25)	118.75(13)	N(3)-C(33)-H(33B)	109.5
C(21)-N(2)-Fe(1)	115.44(9)	H(33A)-C(33)-H(33B)	109.5
C(25)-N(2)-Fe(1)	125.73(10)	N(3)-C(33)-H(33C)	109.5
C(32)-N(3)-C(33)	109.59(11)	H(33A)-C(33)-H(33C)	109.5
C(32)-N(3)-C(34)	109.68(11)	H(33B)-C(33)-H(33C)	109.5
C(33)-N(3)-C(34)	109.86(12)	N(3)-C(34)-C(44)	109.63(11)

C(32)-N(3)-Fe(1)	104.86(8)	N(3)-C(34)-H(34A)	109.7
C(33)-N(3)-Fe(1)	114.27(9)	C(44)-C(34)-H(34A)	109.7
C(34)-N(3)-Fe(1)	108.43(8)	N(3)-C(34)-H(34B)	109.7
C(42)-N(4)-C(43)	108.50(11)	C(44)-C(34)-H(34B)	109.7
C(42)-N(4)-C(44)	109.87(11)	H(34A)-C(34)-H(34B)	108.2
C(43)-N(4)-C(44)	110.33(12)	N(4)-C(42)-C(11)	110.34(11)
C(42)-N(4)-Fe(1)	105.39(8)	N(4)-C(42)-H(42A)	109.6
C(43)-N(4)-Fe(1)	115.17(9)	C(11)-C(42)-H(42A)	109.6
C(44)-N(4)-Fe(1)	107.41(8)	N(4)-C(42)-H(42B)	109.6
N(1)-C(11)-C(12)	122.16(15)	C(11)-C(42)-H(42B)	109.6
N(1)-C(11)-C(42)	116.41(12)	H(42A)-C(42)-H(42B)	108.1
C(12)-C(11)-C(42)	121.42(14)	N(4)-C(43)-H(43A)	109.5
C(13)-C(12)-C(11)	118.77(17)	N(4)-C(43)-H(43B)	109.5
C(13)-C(12)-H(12)	120.6	H(43A)-C(43)-H(43B)	109.5
C(11)-C(12)-H(12)	120.6	N(4)-C(43)-H(43C)	109.5
C(14)-C(13)-C(12)	119.29(16)	H(43A)-C(43)-H(43C)	109.5
C(14)-C(13)-H(13)	120.4	H(43B)-C(43)-H(43C)	109.5
C(12)-C(13)-H(13)	120.4	N(4)-C(44)-C(34)	109.62(11)
C(15)-C(14)-C(13)	119.10(16)	N(4)-C(44)-H(44A)	109.7
C(15)-C(14)-H(14)	120.4	C(34)-C(44)-H(44A)	109.7
N(2)-C(21)-C(32)	115.55(12)	N(4)-C(44)-H(44B)	109.7
C(22)-C(21)-C(32)	122.73(13)	C(34)-C(44)-H(44B)	109.7
C(21)-C(22)-C(23)	119.50(15)	H(44A)-C(44)-H(44B)	108.2
C(22)-C(23)-C(24)	118.92(15)	Cl(23)-Fe(2)-Cl(21)	110.67(2)
C(22)-C(23)-H(23)	120.5	Cl(23)-Fe(2)-Cl(24)	108.94(3)
C(24)-C(23)-H(23)	120.5	Cl(21)-Fe(2)-Cl(24)	109.45(2)
C(25)-C(24)-C(23)	118.96(15)	Cl(23)-Fe(2)-Cl(22)	110.32(2)
C(25)-C(24)-H(24)	120.5	Cl(21)-Fe(2)-Cl(22)	108.41(2)
		Cl(24)-Fe(2)-Cl(22)	109.02(2)

Table S4 - Relevant solid-state NMR data.

	Solvent used for the starting beads					
	EtOH			MeOH		
	**SiO_2**	**SiO_2@CN**	**SiO_2@COOH**	**SiO_2**	**SiO_2@CN**	**SiO_2@COOH**
**^1H MAS**						
	6.1<br>4.7<br>3.5<br>1.12<br>0.05	8.0<br>7.6<br>6.8<br>4.6<br>4.2<br>3.5<br>1.12<br>0.07	7.4<br>4.7<br>4.3<br>3.6<br>1.15	0<br>5.5<br>3.5<br>1.0	7.0<br>4.7<br>3.6<br>1.1	6.0, 6.5<br>3.6<br>1.05
**^{29}Si CP-MAS**						
**T2**		-62.2	-59.6		-64.7	-58.8
**T3**		-70.4	-68.7		-70.5	-68.4
**Q2**	-93.3<br>(5.7)	-92.8<br>(2.2)	-92.8<br>(3.5)	-93.3<br>(5.6)	-93.3<br>(6.3)	-91.9<br>(4.4)
**Q3**	-101.9<br>(58.9)	-101.9<br>(64.5)	-101.9<br>(65.8)	-101.9<br>(55.7)	-102.4<br>(59.8)	-101.8<br>(66.1)
**Q4**	-111.8<br>(35.4)	-111.9<br>(33.3)	-111.7<br>(30.7)	-111.7<br>(38.7)	-111.7<br>33.9)	-111.6<br>(29.5)
**^{29}Si MAS**						
**Q2**	-93.3<br>(8.5)	-92.0<br>(3.4)	-92.5<br>(2.0)	-93.0<br>(6.0)	-93.2<br>(2.5)	-93.7<br>(8.2)
**Q3**	-101.4<br>(30.6)	-101.4<br>(25.6)	-101.4<br>(22.0)	-101.4<br>(28.7)	-102.5<br>(33.0)	-101.9<br>(28.9)
**Q4**	-111.5<br>(60.9)	-111.3<br>(71.0)	-111.6<br>(76.0)	-111.8<br>(65.3)	-111.4<br>(64.5)	-111.5<br>(62.9)
**^{13}C CP MAS**						
**CN**		120.9			121.0	
**COOH**						177.9
**CH_2O**		60.2	60.0			
**CH_2O**	58.0	58.1	58.3	58.2	59.9	59.9
**CH_2O**						49.5
**CH_3**	16.9	16.4	16.6	16.7	16.8	16.7
**CH_2Si**		10.6			10.5	
**CH_2Si**		8.8			8.8	6.7

Table S5 - ^1H NMR chemical shifts (in ppm) observed with **SiO_2**, **SiO_2@CN** and **SiO_2@COOH** in D_2O/NaOH (pH=13) solution.

**S**	**EtOH**		**MeOH**	**$Si(CH_2)_2CN$**		**$Si(CH_2)_2COOH$**	
	CH_2	CH_3	CH_3	CH_2	CH_2	CH_2	CH_2
In EtOH							
**SiO_2**	3.3	0.9	-	-	-	-	-
**SiO_2@CN**	3.4	1.0	-	0.4-0.6	1.9-2.3	-	-
**SiO_2@COOH**	3.3	0.9		-	-	0.4	1.9
**In MeOH**							
**SiO_2**	3.4	0.9	3.1	-	-	-	-
**SiO_2@CN**	3.4	1.0	3.1	0.4-0.6	1.9-2.3	-	-
**SiO_2@COOH**	3.4	1.0	3.1	-	-	0.5	2.0

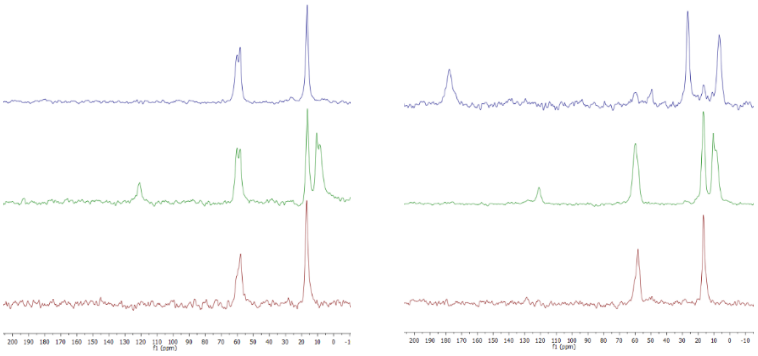

Figure S1 - ^{13}C MAS NMR spectra of **SiO_2** (down), **SiO_2@CN** (middle) and **SiO_2@COOH** (up) for beads from SiO_2 beads done in EtOH (left) and MeOH (right).

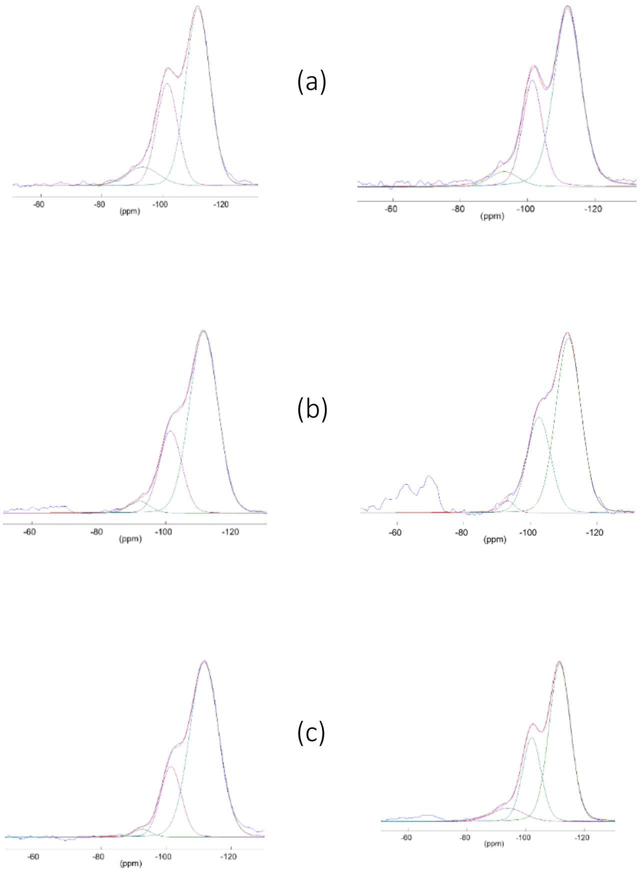

Figure S2 - ^{29}Si MAS NMR spectra of **SiO_2** (up) **SiO_2@CN** (middle), **SiO_2@COOH** (down) from SiO_2 beads done in EtOH (left) and MeOH (right).
